# Typing complex meningococcal vaccines to understand diversity and population structure of key vaccine antigens

**DOI:** 10.12688/wellcomeopenres.14859.2

**Published:** 2019-03-19

**Authors:** Charlene M. C. Rodrigues, Hannah Chan, Caroline Vipond, Keith Jolley, Odile B. Harrison, Jun Wheeler, Gail Whiting, Ian M. Feavers, Martin C. J. Maiden

**Affiliations:** 1 Department of Zoology, University of Oxford, Oxford, OX1 3SY, UK; 2 Division of Bacteriology, National Institute for Biological Standards and Control, Potters Bar, EN6 3QG, UK

**Keywords:** Outer membrane vesicle, OMV, Neisseria meningitidis, meningococcal, vaccines, diversity, proteomics, typing, invasive meningococcal disease

## Abstract

**Background:** Protein-conjugate capsular polysaccharide vaccines can potentially control invasive meningococcal disease (IMD) caused by five (A, C, W, X, Y) of the six IMD-associated serogroups.  Concerns raised by immunological similarity of the serogroup B capsule to human neural cell carbohydrates, meant that ‘serogroup B substitute’ vaccines target more variable subcapsular protein antigens.  A successful approach using outer membrane vesicles (OMVs) as major vaccine components had limited strain coverage. In 4CMenB (Bexsero ^®^), recombinant proteins have been added to ameliorate this problem.

**Methods:** Scalable, portable, genomic techniques were used to investigate the Bexsero ^®^ OMV protein diversity in meningococcal populations. Shotgun proteomics identified 461 proteins in the OMV, defining a complex proteome. Amino acid sequences for the 24 proteins most likely to be involved in cross-protective immune responses were catalogued within the PubMLST.org/neisseria database using a novel OMV peptide Typing (OMVT) scheme.

**Results:** Among these proteins there was variation in the extent of diversity and association with meningococcal lineages, identified as clonal complexes (ccs), ranging from the most conserved peptides (FbpA, NEISp0578, and putative periplasmic protein, NEISp1063) to the most diverse (TbpA, NEISp1690).  There were 1752 unique OMVTs identified amongst 2492/3506 isolates examined by whole-genome sequencing (WGS). These OMVTs were grouped into clusters (sharing ≥18 identical OMVT peptides), with 45.3% of isolates assigned to one of 27 OMVT clusters. OMVTs and OMVT clusters were strongly associated with cc, genogroup, and Bexsero ^®^ antigen variants, demonstrating that combinations of OMV proteins exist in discrete, non-overlapping combinations associated with genogroup and Bexsero ^®^ Antigen Sequence Type. This highly structured population of IMD-associated meningococci is consistent with strain structure models invoking host immune and/or metabolic selection.

**Conclusions:** ****The OMVT scheme facilitates region-specific WGS investigation of meningococcal diversity and is an open-access, portable tool with applications for vaccine development, especially in the choice of antigen combinations, assessment and implementation.

## Introduction

Ideal vaccines comprise a single antigenically conserved component of the target pathogen, preferably against a virulence determinant. The highly successful tetanus and diphtheria toxoid vaccines epitomise this approach, with a major impact on disease globally as part of the World Health Organisation Expanded Programme on Immunisation (WHO EPI) ^1^. Similarly, the conjugate polysaccharide vaccines against *Haemophilus influenzae* type b and certain capsular variants of *Neisseria meningitidis*, the meningococcus, and *Streptococcus pneumoniae*, have been highly successful in eliminating the diseases they cause, largely through herd immunity effects ^2^. A number of more complex vaccines have also been successful, but several of these, including the widely used tuberculosis vaccine BCG ^3^, or those comprising killed whole cells, such as whole cell pertussis and certain typhoid and cholera vaccines, are more difficult to define and control, often achieving imperfect disease control. Antigenically diverse pathogens continue to challenge vaccine design, and it is necessary either: (i) to identify protective antigens that are invariant, as has been attempted with the RTS,S malaria vaccine ^4^, or (ii) to include multiple components, perhaps varying composition over time, as has been done with influenza vaccines over many years.

There is a limited repertoire of meningococcal polysaccharide capsules associated with invasive meningococcal disease (IMD), identified as serogroups A, B, C, W, X, and Y ^5^, potentially simplifying vaccine formulation. While vaccines based on one or a few capsular polysaccharides have been deployed successfully since the 1960s, with notable success achieved by protein-polysaccharide conjugate vaccines ^2^, a comprehensive anti-capsular vaccine has been precluded by concerns about the safety and efficacy of formulations that include the serogroup B polysaccharide. Attempts to generate ‘serogroup B substitute’ vaccines have consequently focussed on subcapsular antigens, especially proteins, but in most cases these are highly variable. Meningococcal diversity extends beyond antigen genes, with a very wide range of distinct genotypes defined by variation in ‘housekeeping genes’ that encode essential cytoplasmic metabolic functions. This diversity is captured by multilocus sequence typing (MLST) approaches, including conventional MLST ^6^, ribosomal MLST (rMLST), and core genome MLST (cgMLST) ^7^. These approaches have shown that both genetic and antigenic diversity of this organism is structured into clusters of related organisms, which share a common ancestor (lineages). These clusters are recognised by conventional seven-locus MLST as clonal complexes (ccs) ^8^. The ccs are correlated with clinical phenotypes, including the propensity to cause disease and its severity ^9^, and antigenic characteristics including capsular and subcapsular antigens ^10^. Comprehensive genomic typing schemes have been developed that catalogue serogroups, ccs and principle vaccine antigens ^11^.

The multiple variants of the subcapsular antigens included in serogroup B substitute vaccines do not necessarily generate cross-reactive protective immune responses against a wide range of distinct meningococci ^12^. Two main approaches have been adopted to overcome this problem: (i) the generation of strain-specific vaccines that target epidemics caused by particular meningococci; (ii) the identification of conserved antigens that offer broad protection. The most widely used strain-specific vaccines have been based on meningococcal outer membrane vesicles (OMVs), which contain outer membrane proteins (OMPs) as principle antigens, especially the PorA porin ^5^. The investigational vaccine ‘Nonamen’ included nine different PorA proteins in a single OMV background to increase population coverage ^13^. ‘Second generation’ meningococcal B substitute vaccine development, using both conventional ^14^ and ‘reverse vaccinology’ ^15^ approaches, identified Factor H binding protein (fHbp) as a leading contender for a single, conserved vaccine antigen, leading to licensure of two vaccines: Bexsero ^®^ (4CMenB) ^16^; and Trumenba ^®^ (rLP2086) ^14^. The fHbp protein is, however, highly variable in meningococcal populations ^17^, so the effectiveness of the protection offered, not fully established at the time of writing, is dependent on the generation of cross-protective immune responses ^12^.

The bivalent Trumenba ^®^ vaccine, containing two lipidated fHbp variants was licenced for use in the US in 10–15-year-olds in 2014 and for those aged 10 years or over in Europe in 2017; however, as of summer 2018, Bexsero ^®^ vaccine was the only serogroup B substitute vaccine licenced, in 2013, for use in infants. This vaccine was introduced into the UK infant immunisation programme in 2015 ^18^, undergoing post implementation assessment ^19^ and comprising three recombinant meningococcal proteins: fHbp, neisserial heparin binding antigen (NHBA); and *Neisseria* adhesin A (NadA); together with the MeNZB™ OMV that contained PorA as a principal antigen ^20^. The meningococcal antigen typing system (MATS) immunological assay had been used to assess potential cross-reactivity of toddler antibodies to the principal Bexsero ^®^ components ^21, 22^ and a Bexsero ^®^ Antigen Sequence Typing scheme (BAST) was developed to assess the sequence variability of these vaccine components from meningococcal whole-genome sequence (WGS) data ^23, 24^. At the time of writing, however, no means were available to systematically asses the other protein components of the OMV, which could potentially contribute to cross-immunity generated by this vaccine. Shotgun proteomics was performed to identify the protein content of the Bexsero ^®^-derived OMVs, and alongside published data ^25^, informed the selection of proteins for use in the development of an Outer Membrane Vesicle peptide Typing (OMVT) scheme. Here we describe the OMVT scheme and investigate its value in supporting the assessment of vaccine impact and the improvement of vaccine formulation.

## Methods

### Proteomic OMV sample preparation

Two batches of meningococcal NZ98/254 derived OMVs, from GlaxoSmithKline, Siena Italy, were analysed in triplicate. Each replicate contained 100 µg total protein in 0.5% SDS and 200 mM triethylammonium bicarbonate, (TEAB), the pH 8.0 was reduced with tris(2-carboxyethyl) phosphine and alkylated with iodoacetamide before an overnight acetone precipitation. A 200 mM TEAB solution containing 2.5 µg trypsin (Promega) was directly added to the protein pellet and digestion was performed at 37°C overnight. The resulting peptides from each sample in a set were labelled with a different isobaric tag (TMTs 126–131, ThermoFisher) before being mixed into a single sixplex sample.

A sample of labelled peptide mixture was injected on to a XBridge C18 column, (5 µm, 4.6 mm id and 25 cm long, Waters) for the first-dimension high pH RP-HPLC separation under a linear gradient consisting mobile phase A (10 mM ammonium formate, pH 10.0) and up to 70% B (90% acetonitrile in mobile phase A) for 2 hours at flow rate of 0.5 ml/min, using a Jasco system consisting an autosampler, semi-micro HPLC pumps and UV detector. Eluted fractions were collected and concatenated into eight tubes and vacuum dried. As OMPs are not all equally susceptible to enzymatic digestion, we evaluated different sample preparation methods. The method described provided an increased number of OMPs to other studies in our laboratory and the contribution of membrane to cytoplasmic proteins was comparable to other studies ^25– 27^.

### Nano-LC-MS/MS

Nano-LC and tandem mass spectrometry (MS/MS) was performed using a U3000 direct nano system coupled with nano-electrospray and LTQ-Orbitrap Discovery mass spectrometer (Thermo). Resuspending in 0.1% formic acid, the HPLC fractions containing a mixture of sixplex labelled peptides were separated on a PepMap C18 reversed phase nano column (3 µm, 100 Ǻ, 50 cm length, Thermo) under a column flow rate of 0.3 µl/min using linear gradient of 5–25% for 180 min, 25–32% for 20 min and 32–90% for 10 min of 95% acetonitrile and 0.1% formic acid. MS scan and MS/MS fragmentation were carried out in Orbitrap and LTQ mass analysers, respectively, using 2 cycles of top 3 data-dependent acquisition with dynamic exclusion mode enabled and total cycle time at approximately 30 milliseconds. The first cycle used collision-induced dissociation (CID) fragmentation generating spectra for peptide sequencing, and the second High energy CID (HCD).

### Proteomic data analysis

Mass spectra processing, database searching and quantitation were carried out using Thermo Proteome Discoverer 1.4 with built-in Sequest against the Uniprot *N. meningitidis* MC58 FASTA database, release 2014_03. Spectra from the 8 fractions were added together as one sample during searching. Initial mass tolerances by MS were set to 10 ppm. Up to two missed tryptic cleavages were considered. Methionine oxidation was set as dynamic modification whereas carboxymethylation on cysteine and TMT6plex labels on N-terminal amino acid and lysine side chain were set as static modifications. Positive protein identification was considered when matched to minimum of two peptides sequenced at rank 1 with high confidence. Protein FASTA sequences identified were subsequently submitted to a web server for protein subCELular LOcalisation prediction ( http://cello.life.nctu.edu.tw/cello2go/) and PSORTb; Supplementary Table 1) ^28– 30^.

### OMVT scheme development and nomenclature

**Figure 1.**
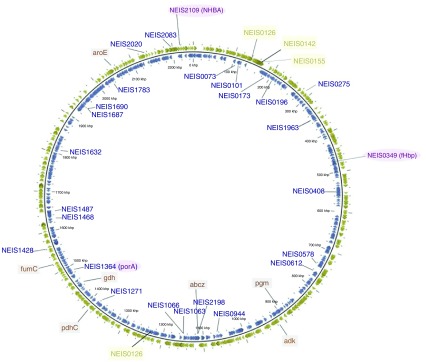
Representation of *Neisseria meningitidis* reference genome NZ05/33, drawn with Gview ^32^. NZ05/33 is an ST41/44 outbreak strain from New Zealand. The MeNZB™ vaccine was developed from NZ98/254, another ST41/44 isolate from the same New Zealand outbreak. Chromosomal location of the genes encoding the proteins included in the Outer Membrane Vesicle peptide Typing (OMVT) scheme are shown in blue. Genes encoding four cytoplasmic proteins are shown in yellow boxes. The multilocus sequence typing loci are shown in brown boxes. The genes encoding the constituents of the Bexsero ^®^ Antigen Sequence Typing scheme are shown in purple ellipses, note that *nadA* is absent in this strain.

A total of 25 proteins were chosen, primarily for pragmatic reasons as this was considered manageable for detailed curation within the typing scheme. The proteins represented the 20 most abundant outer membrane or periplasmic proteins identified in by Nano-LC-MS/MS with an additional five proteins chosen from the published core proteome ^25^, also identified by Nano-LC-MS/MS ( Table 1). All proteins chosen for the scheme were predicted to be either outer membrane or periplasmic and therefore more likely to be OMV antigens than the cytoplasmic proteins, which were more abundant in the proteomic analysis. The cytoplasmic proteins are unlikely to be constituents of the meningococcal outer membrane and their presence in OMV samples is a probable consequence of cell lysis during detergent extraction causing cytosolic proteins to be released into the preparations ^31^. Although the Pilin E (PilE) protein was amongst this group, it was excluded from the scheme due to high frequency (>60%) of sequencing or assembly difficulties, which were a consequence of the highly repetitive DNA regions and high diversity of this antigen, with multiple copies in the meningococcal genome. Thus, 24 proteins were taken forward and defined using a novel peptide-sequence based nomenclature and visualised with GView (Version 1.7) ( Figure 1) ^32^. Each of the 24 peptides was been given the prefix NEISp, referring to the peptide sequence deduced from the corresponding nucleotide locus, designated with the prefix ‘NEIS’. Each peptide was numbered according to its equivalent NEIS number. For example, *porA* has a nucleotide locus NEIS1364, and peptide locus NEISp1364. This collection of 24 peptide antigens form the OMVT scheme, which is hosted on the PubMLST *Neisseria* database ^33^. The closed reference genome from New Zealand isolate NZ05/33 was used to identify variant 1 amino acid sequence for each peptide included in the OMVT scheme. This isolate shares 99.4% sequence identity and 1595/1605 core genome loci with NZ98/254, the New Zealand outbreak isolate used to make the MeNZB™ vaccine. Every unique amino acid sequence variant subsequently identified was assigned a unique variant number, in order of discovery.

**Table 1.** Details of the outer membrane vesicle (OMV) proteins included in the Outer Membrane Vesicle peptide Typing (OMVT) scheme including the corresponding NEISp number assigned with respect to the NEIS nucleotide sequence on PubMLST.org.

NEISp annotation	NEIS no. (Gene number)	UniProtKB identifier	Cellular location	Protein description	Isolates with peptide variant present n (%)	Isolates with null peptide variant (0) n (%)
0073	NEIS0073 (NMB0088)	A0A0E0TMZ0	OM	Outer membrane protein, OmpP1 (OmpP1/FadL/ TodX family)	3438 (98.06)	64 (1.83)
0101	NEIS0101 (NMB0109b)	A0A0E0TRC4	Unknown	LysM domain/M23 peptidase domain protein	3506 (100.00)	0 (0.00)
0173	NEIS0173 (NMB0182)	A0A0E0TN68	OM	Outer membrane protein assembly factor BamA (Omp85)	3501 (99.86)	0 (0.00)
0196	NEIS0196 (NMB0204)	A0A0E0TN82	OM	Outer membrane protein assembly factor BamE (Lipoprotein)	3504 (99.94)	0 (0.00)
0275	NEIS 0275 (NMB0280)	F0MIT7 (H44/76)	OM	LPS-assembly protein LptD / Putative organic solvent tolerance protein	3442 (98.17)	4 (0.11)
0408	NEIS0408 (NMB1812)	A0A0E0TNY5	OM	Type IV pilus secretin PilQ (PilQ)	3429 (97.80)	36 (1.03)
0578	NEIS0578 (NMB0634)	A0A0E0TPP5	PP	Iron (III) ABC transporter, periplasmic iron (III)- binding protein (FbpA)	3501 (99.86)	0 (0.00)
0612	NEIS0612 (NMB0663)	A0A0E0TQI3	OM	Surface protein A (NspA)	3498 (99.77)	0 (0.00)
0944	NEIS0944 (NMB0964b)	A0A0E0TQ95	OM	Iron receptor protein (Irp, TonB-dependent receptor)	3203 (91.36)	64 (1.83)
1063	NEIS1063 (NMB1124/ NMB1162)	A0A0E0TQ69	OM	Putative lipoprotein / Putative periplasmic protein	3451 (98.43)	6 (0.17)
1066	NEIS1066 (NMB1126/1164)	A0A0E0TQU6	PP	CsgG family protein / Putative lipoprotein	3459 (98.66)	40 (1.14)
1271	NEIS1271 (NMB1333)	A0A0E0TRB5	OM	M23 peptidase domain protein	3471 (99.00)	3 (0.09)
1364	NEIS1364 (NMB1429)	A0A0E0TSG1	OM	PorA (Outer membrane protein IA)	3400 (96.98)	52 (1.48)
1428	NEIS1428 (NMB1497)	A0A0E0TR48	OM	Heme-utilization protein (Hup, TonB-dependent receptor)	3436 (98.00)	6 (0.17)
1468	NEIS1468 (NMB1540)	A0A0E0TRH9	OM	Lactoferrin-binding protein A (LbpA)	3240 (92.41)	43 (1.23)
1487	NEIS1487 (NMB1567)	A0A0E0TRL0	OM	Macrophage infectivity potentiator (Peptidyl- prolyl cis-trans i Peptidyl-prolyl cis-trans isomerasesomerase, Mip/FkpA)	3504 (99.94)	0 (0.00)
1632	NEIS1632 (NMB1714)	A0A0E0TRR1	OM	Multiple transferable resistance system protein (Multidrug efflux pump channel protein, MtrE)	3501 (99.86)	4 (0.11)
1687	NEIS1687 (NMB0464)	A0A0E0TTD6	OM	Phospholipase A1 (PldA)	2732 (77.92)	729 (20.79)
1690	NEIS1690 (NMB0461)	A0A0E0TSK9	OM	Transferrin-binding protein 1 (TbpA)	3268 (93.21)	2 (0.06)
1783	NEIS1783 (NMB0382)	A0A0E0TTL2	OM	Outer membrane protein class 4 (OmpA family protein, RmpM)	3505 (99.97)	0 (0.00)
1963	NEIS1963 (NMB1988)	A0A0E0TNL5	OM	Enterobactin receptor FetA (FetA)	3384 (96.52)	52 (1.48)
2020	NEIS2020 (NMB2039)	A0A0E0TSH4	OM	Major outer membrane protein PIB (PorB)	3468 (98.92)	1 (0.03)
2083	NEIS2083 (NMB0375)	A0A0E0TU30	OM	Adhesin MafA family (Putative lipoprotein MafA)	3046 (86.88)	194 (5.53)
2198	NEIS2198 (NMB1053)	A0A0E0TQB2	OM	Class 5 outer membrane protein (OpcA)	2217 (63.23)	1282 (36.57)

EC, extracellular; IM, inner membrane; PP, periplasmic.

### Peptide variant designation and caveats

For each component of the OMVT scheme, peptide variants were assigned from deduced peptide sequences of the respective NEIS locus. Where the NEIS locus was a complete coding sequence, i.e. containing a start and terminal stop codons with the number of base pairs (bp) being a multiple of three and no internal stop codons, each unique peptide sequence was assigned a unique arbitrary variant number in order of identification. Where the gene contained a mutation generating a frameshift, indel, or an internal stop codon more than 50 bp from the consensus stop codon, then variant 0 (null) was assigned, following the deduction that no functional protein would be produced from such loci. Where internal stop codons occurred fewer than 50 bp from the consensus stop codon, the peptide variants were assigned. Where no NEIS or NEISp variant could be deduced, due to incomplete sequencing or genome assembly, no variant was assigned.

### OMVTs

For OMVT profiles with all 24 loci present, each unique combination was assigned an integer, the OMVT. For example, OMVT-1 corresponded to the OMV peptide variants present in the NZ05/33 genome. All subsequent OMVTs were numbered in order of identification. Although OMVTs provided a rapid means of identifying isolates with identical profiles, there were related isolates that differed at ≤23 loci. To analyse these relationships among OMVTs, a clustering method was applied using eBURSTv3, which determined non-overlapping groups of closely-related strains, i.e. no OMVT was allocated to more than one OMVT cluster, and entry into a cluster required matching at ≥18 loci to the central OMVT ^34^. To ensure stability of OMVT clusters, a central OMVT was defined as the OMVT that differed from the largest number of other OMVTs at only a single peptide sequence. This was also used to name the cluster, for example OMVT-449 was the central OMVT of the OMVT-449 cluster. Bootstrapping for each group was assessed using the 23-peptide cut-off.

The OMVT scheme was used to catalogue the diversity of IMD isolates from the Meningitis Research Foundation Meningococcus Genome Library (MRF-MGL) and related isolate collections. This included 3506 WGS of all culture-confirmed meningococcal isolates in epidemiological years 2010/11 to 2016/17 from England, Wales, Scotland, and Northern Ireland, representing approximately 55% of all laboratory-confirmed IMD in these regions ^24, 33^. All isolates in the collection were automatically annotated for the OMVT variants, with manual curation of new variants. OMVTs and OMVT clusters were assigned accordingly.

### Cluster analysis

GrapeTree software ^35^ was used to cluster allelic profiles from large collections of WGS and visualise these relationships. It provides an interactive, web-based interface associated with relevant metadata including: year, serogroup, cc, and BAST. GrapeTree was run through the PubMLST.org plug-in.

### Statistical analysis

All statistical analyses were performed using R version 3.2.4. Cramer’s V coefficient was used to assess the association of peptide loci with cc and was calculated using the ‘Cramer’s V’ function in the ‘ lsr’ package (v0.5) in R. The diversity of each OMVT protein was assessed using Shannon’s index of diversity (H) and the “adjusted diversity” calculated from the natural log of the number of peptide variants/amino acid/isolate. H was calculated using the ‘ Vegan’ package (v2.4-v2.6) in R, H represents the uncertainty in predicting the peptide variant of a new isolate, given the number of peptide variants and the evenness in abundance of isolates possessing each variant. H increases as richness (number of variants) and evenness (distribution) in the population increases.

## Results

### The proteome of the OMV from Bexsero ^®^

**Figure 2.**
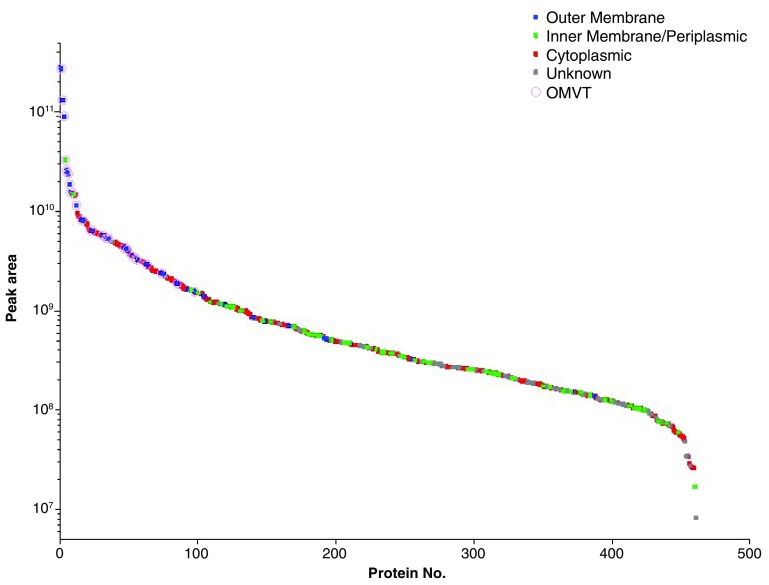
Relative amount of protein as determined from peak area of tandem mass spectrometry data. Proteins were assigned to a group using CELLO ^29, 30^ and PSORTb ^28^ informatic tools, where the predictions differed proteins were analysed manually using UNIPROT and available published data to define a location. Where a location could not be predicted proteins were assigned an unknown status.

A total of 461 proteins, representing the proteome of the NZ98/254 OMV, were unambiguously identified in this study ( Supplementary Table 1). The predicted subcellular location of each protein revealed 282 cytoplasmic proteins, 60 inner membrane or periplasmic proteins, 36 outer membrane or extracellular proteins and 83 that could not be definitively assigned to any location. Other proteomic analysis of OMVs have found between 100-300 proteins ^25– 27^, with an increasing number of proteins identified in the OMVs presumably due to technical advances improving the sensitivity of mass spectrometers. All studies share the finding that membrane and periplasmic proteins dominate the proteome in terms of abundance. The peak areas of the top three most abundant peptides were used to provide a relative quantity of each of the proteins. By summing the peak areas of all the proteins from each location, the protein abundance in each sub-cellular location was determined with 58% of the total protein from the outer membrane, 6% from either the inner membrane or periplasm, 32% cytoplasmic proteins, and 3% without a location defined. The 24 proteins chosen for the OMVT scheme encompass 60% of the total protein found in the OMVs ( Figure 2).

### Peptide variants in OMVT scheme

**Figure 3.**
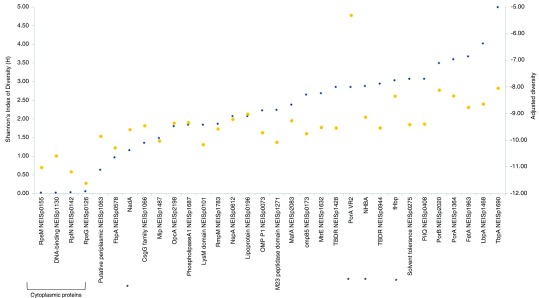
Diversity of each protein in the Outer Membrane Vesicle peptide Typing scheme. Diversity is measured by Shannon’s index of diversity (H) for each peptide locus (indicated by the dark blue dots and left axis) and adjusted diversity (AD), the natural log transformation of the number of variants per amino acid per isolate (indicated by yellow dots and right axis). All data were deduced from a dataset of 3506 UK invasive meningococcal disease isolates. Four cytoplasmic proteins are shown on the left of the x axis for reference, three of which are ribosomal proteins RpsM, RplN, RpsG known to be highly conserved in bacteria. The recombinant proteins in the Bexsero ^®^ vaccine are marked with an *, factor-H binding proteins (fHbp), Neisserial Heparin binding antigen (NHBA), *Neisseria* adhesin A (NadA) and Porin A variable region 2 (PorA VR2).

Peptide variants (including null, 0) were annotated in ≥98% of the WGS data of the 3506 UK isolates. As a consequence of incomplete gene assembly, annotation was lower for: Irp (NEISp0944), 3267/3506 isolates (93.2%); LbpA (NEISp1468), 3283/3506 (93.6%); TbpA (NEISp1690), 3270/3506 (93.3%); and MafA (NEISp2083), 3240/3506 (92.4%), ( Table 1). H was used to assess the richness and evenness of variants, with TbpA the most diverse antigen (H=3.94) and putative periplasmic protein NEISp1063 the least diverse (H=1.04) ( Figure 3, Table 2). The OMPs were more diverse than cytoplasmic proteins (p<0.00001). The adjusted diversity for each locus ranged from -10.3 for FbpA (NEISp0578) to -8.1 for TbpA (NEISp1690) and PorB (NEISp2020) ( Figure 3, Table 2).

**Table 2.** Outer Membrane Vesicle peptide Typing (OMVT) scheme association with clonal complex and peptide diversity. Association between individual outer membrane vesicle proteins included in the OMVT scheme and clonal complex (cc) as measured by the Cramer’s V statistic, where values approaching one support an association. Measures of diversity of each locus are given as Shannon’s index of diversity and adjusted diversity.

OMVT proteins	Cramer’s V	Shannon’s index of diversity	Adjusted diversity
Irp (NEISp0944)	0.92	2.84	-9.62
Hup (NEISp1428)	0.92	2.62	-9.55
TbpA (NEISp1690)	0.90	3.94	-8.12
FetA (NEISp1963)	0.89	3.01	-8.80
LbpA (NEISp1468)	0.87	3.40	-8.72
PorA (NEISp1364)	0.83	3.19	-8.37
LptD (NEISp0275)	0.80	3.11	-9.43
PorB (NEISp2020)	0.77	2.86	-8.12
OmpP1 (NEISp0073)	0.74	2.33	-9.74
MtrE (NEISp1632)	0.73	2.46	-9.51
OMP85 (NEISp0173)	0.69	2.71	-9.89
PilQ (NEISp0408)	0.67	3.11	-9.43
MafA (NEISp2083)	0.63	2.16	-9.41
M23 peptidase domain protein (NEISp1271)	0.60	2.22	-10.08
OpcA (NEISp2198)	0.58	1.77	-9.81
NspA (NEISp0612)	0.57	2.17	-9.21
Lipoprotein (NEISp0196)	0.52	2.16	-9.02
FbpA (NEISp0578)	0.44	1.23	-10.28
Mip/FkpA (NEISp1487)	0.41	1.65	-10.03
PldA (NEISp1687)	0.41	1.95	-9.59
RmpM (NEISp1783)	0.41	1.87	-9.57
LysM domain protein (NEISp0101)	0.41	1.92	-10.16
CsgG family protein (NEISp1066)	0.41	1.62	-9.46
Putative periplasmic protein (NEISp1063)	0.34	1.04	-9.85

**Figure 4.**
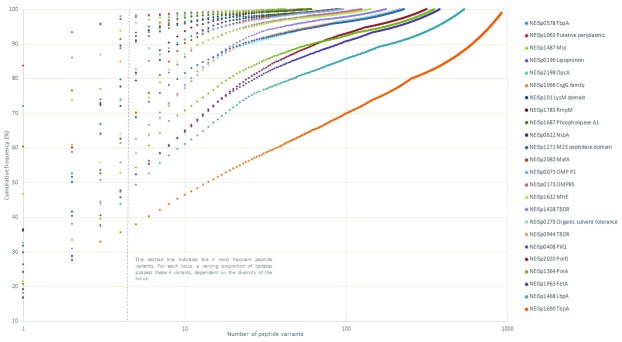
Distribution of Outer Membrane Vesicle peptide Typing (OMVT) variants in UK meningococcal disease isolates. The cumulative frequency distribution is shown for each locus in the OMVT scheme. For each peptide, it shows the proportion of the 3506 isolates that were annotated with each successive peptide variant. The grey dashed line indicates the proportion of isolates within the dataset which possess the four most frequent variants. The distribution is highly skewed, as for FbpA (NEISp0578), Putative periplasmic protein (NEISp1063), Mip/FkpA (NEISp1487) and PldA (NEISp1687) four variants annotated >90% of isolates, contrasting with TbpA for which only 35.6% of isolates possessed the four most frequent peptide variants.

**Figure 5.**
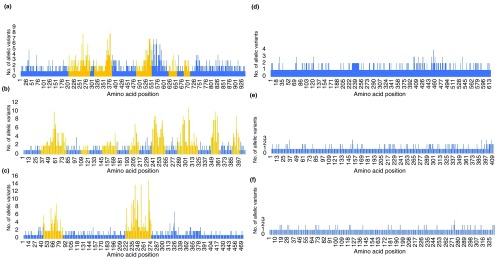
Variation in the diversity across the amino acid sequence for outer membrane proteins. The number of allelic variants for each position of the protein are shown for the three most diverse proteins on the left ( **a**) TbpA (NEISp1690), ( **b**) PorB (NEISp2020), ( **c**) PorA (NEISp1364) and the three most conserved ( **d**) M23 peptidase domain protein (NEISp1271), ( **e**) Putative peptidoglycan-binding periplasmic protein, LysM domain-containing protein (NEISp0101), ( **f**) FbpA (NEISp0578). Variable regions are shown in yellow for TbpA, PorB and PorA, as previously described ^35– 37^. There were other proteins that had potential variable regions including well-described protein FetA (NEISp1963), LbpA (NEISp1468) and Ton-B dependent receptor (NEISp1428).

The distribution of OMVT protein variants within the UK IMD isolates was non-random. A cumulative frequency distribution of the peptide variants for each locus, for the 3506 IMD isolates, demonstrated the loci for which four variants are found in >90% of isolates including: FbpA (NEISp0578); putative periplasmic protein (NEISp1063); Mip/FkpA (NEISp1487); and PldA (NEISp1687) ( Figure 4). Other loci, including TbpA, LbpA, FetA, PorA, and PorB, showed more diversity and variants were distributed more evenly across the 3506 isolates. The distribution of the variation within each protein was also non-random, i.e. the variation was not distributed evenly across the amino acid sequence. In many OMVT proteins there were regions of high diversity concentrated in the so-called “variable regions”, representing surface-exposed loops under immune selection, previously well-characterised for TbpA, PorB, and PorA ^36– 38^ ( Figure 5).

### Distribution of OMVTs in IMD and association with cc

**Figure 6.**
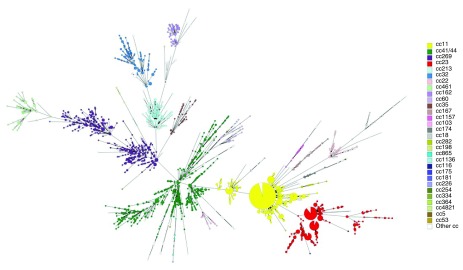
UK meningococcal disease isolates clustered by the Outer Membrane Vesicle peptide Typing (OMVT) scheme. Invasive meningococcal disease isolates from epidemiological years 2010 to 2017 (n=3506) analysed using the 24 loci OMVT scheme and visualised using GrapeTree software. The OMVT cluster by clonal complex (cc), which are shown by different colours, with unfilled nodes representing isolates from the other ccs. The size of the circles is proportional to the number of isolates.

The OMVT scheme was applied to the WGS data from the 3506 UK meningococcal isolates. A total of 1752 OMVTs were assigned to 2492/3506 (71.1%) isolates. The remaining 1014 (28.9%) isolates had one or more incomplete OMVT protein-encoding loci and it was not possible to assign an OMVT. The most frequently occurring type was OMVT-1149 in 246/2492 (9.9%) isolates, which was associated with cc11 genogroup W isolates (W:cc11). The most frequent OMVTs represented by 10 or more isolates were: OMVT-21, 64/2492 (2.6%); OMVT-50, 32/2492 (1.3%); OMVT-1275, 18/2492 (0.7%); OMVT-1161, 16/2492 (0.6%,); OMVT-1199 16/2492 (0.6%); and OMVT-1158, 12/2492 (0.5%). There were: 1547 OMVTs that were represented by only one isolate (1547/2492 (62.1%); 133 represented by 2 isolates (266/2492, 10.7%); and 65 represented by 3–10 isolates (275/2492, 11.0%). Analysis of all isolates for which an OMVT was available (71.1%, 2492/3506), demonstrated that OMVT was strongly associated with cc: Cramer’s V=1, with values closer to 1 representing strong association ( Figure 6) ^35^. To determine whether a different subset of OMV proteins could alter the clustering of isolates, the same 3506 meningococcal genomes were analysed using 50 OMV proteins (OMPs, periplasmic and cytoplasmic), showing similarly strong association with clonal complex (data not shown). To determine the extent of association of individual OMVT proteins with cc, Cramer’s V was calculated for each protein. The values ranged from 0.92 for two TonB-dependent receptors (NEISp0944 and NEISp1428) to 0.34 for the putative periplasmic protein (NEISp1063) ( Table 2), suggesting that OMVT proteins with lower Cramer’s V were found across ccs.

### OMVT clusters and association with BAST

**Figure 7.**
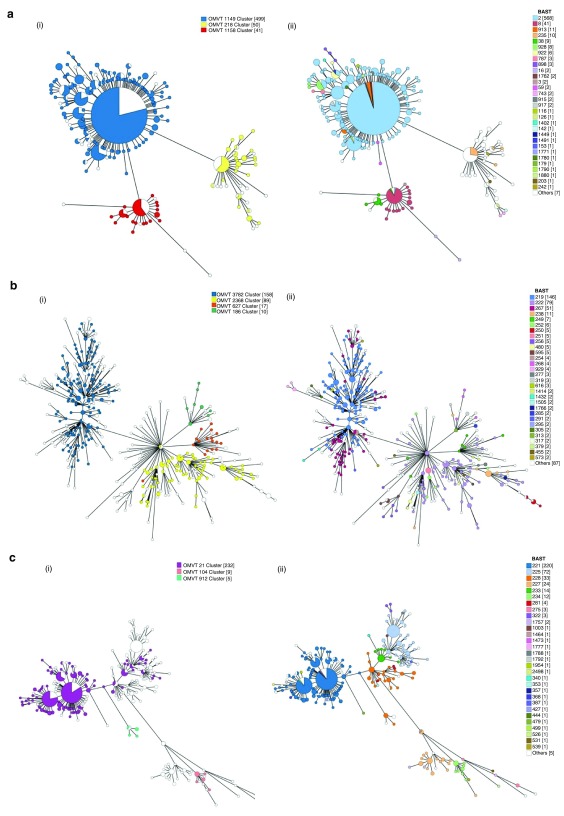
Outer Membrane Vesicle peptide Typing (OMVT) clusters and association with Bexsero ^® ^Antigen Sequence Types (BASTs). ( **a**) Clonal complex 11 (cc11) isolates (n=777) were analysed using the OMVT scheme. (i) Clusters of OMVTs that match 18 loci to the central OMVT are shown. Cc11 has 3 OMVT clusters, 1149, 218 and 1158. OMVT 1149 cluster represents lineage 11.2, OMVT clusters 218 (yellow) and 1158 (red) represent lineage 11.1. (ii) OMVT clusters are coloured by BASTs, demonstrating the strong non-overlapping association of BASTs with OMVT clusters. Unfilled nodes represent incomplete data, so a profile could not be assigned. ( **b**) Cc269 isolates (n=500) were analysed using the OMVT scheme. (i) Cc269 has 4 OMVT clusters. (ii) OMVT clusters are coloured by BAST. ( **c**) Cc23 isolates (n=459) were analysed using the OMVT scheme. (i). Cc23 has 3 OMVT clusters. (ii) OMVT clusters are coloured by BAST.

Given the large number of possible OMVTs, they were grouped into OMVT clusters, which differed by up to six loci from a defined central OMVT, i.e. isolates in OMVT-1149 cluster were identical to OMV-1149 for ≥18 loci. There were 1587/3506 (45.3%) isolates assigned to 27 OMVT clusters. Each OMVT cluster was predominantly associated with a cc and a genogroup ( Table 3). The recombinant Bexsero ^®^ antigens had a strong association with the OMVTs, as measured by Cramer’s V (fHbp=0.98; NHBA=0.99; NadA=0.96; PorAVR1=1; PorAVR2=1) and OMVT clusters were also associated with BAST (Cramer’s V = 0.96) ( Table 3) ^23^. These associations were visualised with the GrapeTree clustering algorithm for OMVT clusters and BAST for cc11, cc269, and cc23 ( Figure 7a–c) ^35^. Some large ccs exhibited a degree of subtracting; for example, cc11 exhibited three OMVT clusters (OMVT-1149 cluster, OMVT-218 cluster and OMVT-1158 cluster). Each OMVT cluster exhibited a non-overlapping repertoire of BASTs.

**Table 3.** OMVT clusters were associated with clonal complex, genogroup and Bexsero ^®^ Antigen Sequence Type (BAST), comprised of the other recombinant proteins found in the 4CMenB vaccine Bexsero ^®^.

OMVT cluster	No. of isolates	Clonal complex	Genogroup	% with genogroup	BASTs
OMVT 104 Cluster	10	32	Y	100	234, 539
OMVT 1142 Cluster	14	23	B	100	4, 19
OMVT 1149 Cluster	502	11	W	99.8	2, 3, 50, 59, 68, 116, 153, 179, 787, 820, 898, 913, 915, 917, 922, 928, 1402, 1491, 1762, 1780, 1880
OMVT 1152 Cluster	26	32	B	100	5, 61, 62, 108, 110, 138, 155, 391, 535, 925, 1397, 1419, 1701, 1878
OMVT 1158 Cluster	42	11	C	97.5	3, 8, 38
OMVT 1229 Cluster	4	32	B	100	309
OMVT 1247 Cluster	23	461	B	100	230, 296, 307, 407, 520, 525, 543, 571, 587, 1009, 1800
OMVT 1256 Cluster	12	32	B	100	56, 88, 160, 195, 618, 918, 926, 1773, 2138
OMVT 1316 Cluster	29	162	B	100	244, 246, 286, 326, 374, 502, 568, 807, 873
OMVT 1341 Cluster	16	32	B	100	10, 178, 1764
OMVT 151 Cluster	9	35	B	100	290, 445
OMVT 160 Cluster	67	41/44	B	100	220, 223, 226, 229, 231, 236, 239, 280, 287, 312, 316, 347, 438, 448, 515
OMVT 186 Cluster	10	269	B	100	238, 268, 313, 319, 591
OMVT 21 Cluster	240	23	Y	99.1	221, 225, 228, 233, 357, 368, 387, 427, 444, 499, 579, 628, 964, 1473, 1792, 1824, 1954, 2498
OMVT 211 Cluster	5	35	B	100	257, 293, 320
OMVT 218 Cluster	53	11	C	84.3	126, 142, 235, 242, 521, 522, 546
OMVT 257 Cluster	4	41/44	B	100	220
OMVT 27 Cluster	91	213	B	100	224, 245, 258, 264,304, 310, 324, 328, 333, 335, 336, 346, 356, 369, 383, 395, 400, 403, 417, 471, 504, 509, 517, 518, 537, 552, 556, 608, 617, 623, 634, 2879, 2882
OMVT 368 Cluster	91	269	B	100	222, 238, 249, 251, 252, 277, 358, 379, 455, 468, 480, 501, 550, 595, 602, 668, 973, 1413, 1505, 1766
OMVT 3782 Cluster	196	269	B	100	219, 254, 267, 273, 291, 295, 431, 446, 452, 460, 473, 524, 536, 583, 598, 611, 616, 624, 633, 942, 946, 947, 953, 1273, 1424, 1816
OMVT 414 Cluster	34	41/44	B	100	220, 223, 226, 240, 260, 278, 562, 589, 2874
OMVT 449 Cluster	47	22	B	95.5	265, 300, 301, 330, 349, 386, 429, 440, 466, 1765, 1776
OMVT 627 Cluster	17	269	B	62.5	222, 249, 360, 405, 423, 485, 491, 510, 604, 1414, 1821
OMVT 654 Cluster	11	103	C	72.7	288, 311, 541, 544, 553, 566
OMVT 905 Cluster	13	167	Y	100	261, 461, 577, 1406, 1805
OMVT 912 Cluster	5	23	Y	100	228
OMVT 932 Cluster	16	174	Y	80	11, 14, 72, 117, 127, 135, 146, 177

### Distribution of OMVT variants by OMV vaccine

**Figure 8.**
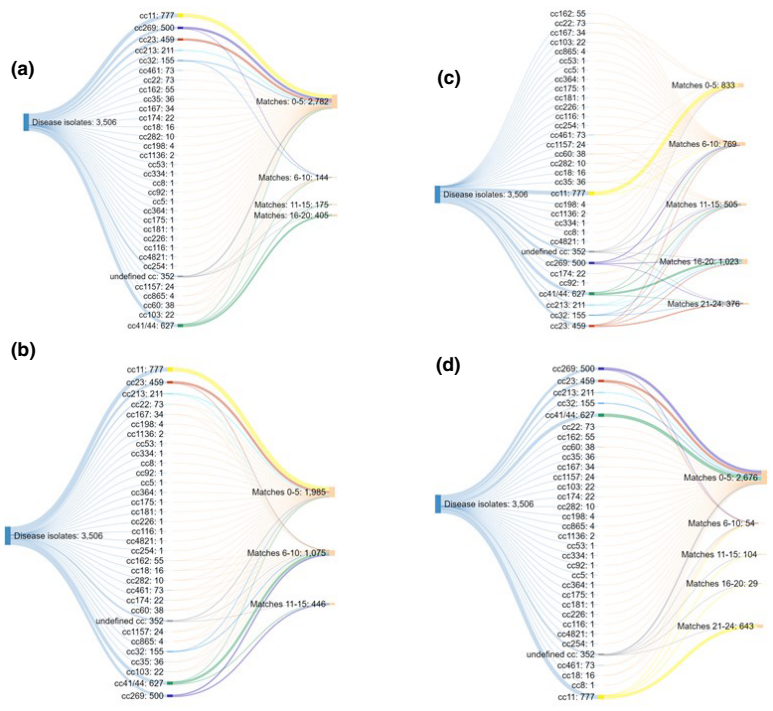
The distribution of UK meningococcal disease isolates that match the peptide variants in different vaccine formulations. Isolates were scored at each of the 24 loci that shared peptide sequence variants with real ( **a**) and hypothetical vaccine formulations ( **b**– **d**). ( **a**) OMV vaccine MeNZB™ clonal complex 41/44 (cc41/44), ( **b**) selected OMVT protein variants that occur at high frequency in this dataset, ( **c**) engineered OMV including multiple OMVTs (-160, -368, -1152, -27, -21), ( **d**) OMVT-1149 based vaccine (cc11). The disease isolates (n=3506) were stratified by cc (n shown after each cc) and then the number of matches, 1-24, are displayed on the right (n shown after the number of matches). The thickness of the lines is proportional to the number of isolates. Undefined cc represents STs that have not been assigned to a cc or isolates that had a partial multilocus sequence typing profile.

OMV vaccines can be uniquely identified using OMVTs; for example MeNZB™ and Bexsero ^®^ vaccines are OMVT-1. Each OMVT deduced protein from all 3506 isolates were compared to the variants present in OMVT-1, a perfect match giving a score of 24. Only 10/3506 (0.3%) of isolates did not share any peptide variants with MeNZB™, these belonged to cc23, cc213, and cc116. The only isolates with a defined cc that had 11 or more shared variants with MeNZB™ belonged to cc41/44 ( Figure 8a). There were 34 isolates with undefined cc with ≥11 shared variants with MeNZB™, corresponding to STs that have not been assigned to a cc or isolates that had a partial MLST profile related to cc41/44. The cc41/44 isolates comprised 169 STs. Among these cc41/44 isolates, there was a bimodal distribution of OMVT variants shared with MeNZB™, suggesting two distinct groups: ST-41, ST-1194, ST-485, ST-340, ST-154, and ST-2314 isolates had ≥12 shared variants, whereas ST-1097, ST-409, ST-6508, and ST-571 isolates had ≤10 shared variants with MeNZB™. There were 2772/3506 (79%) isolates that shared 1–5 variants with MeNZB™, this was due to the presence of identical variants of: FbpA (NEISp0578); NspA (NEISp0612); putative periplasmic protein (NEISp1063); CsgG family protein (NEISp1066); and Mip/FkpA (NEISp1487). These were found in a high proportion of isolates, independent of cc: NEISp0578 2524/3506 (72.0%); NEISp0612, 1275/3506 (36.4%); NEISp1063 2932/3506 (83.6%); NEISp1066 2115/3506 (60.3%); and NEISp1487 1637/3506 (46.7%).

The same approach was used to analyse other OMV vaccines: MenBVac ^®^; MENGOCOC-VA ^®^; and the Chilean OMV vaccine, all of which were derived from cc32 isolates and optimised OMV formulations ( Figure 8b, c). By identifying variants that occurred at high frequency and across the most prevalent OMVT clusters, the composition of the 24 OMVT proteins was manipulated to improve the number of antigenic matches amongst the set of UK disease isolates. Devising a formulation comprising the protein variants found in the most frequently occurring ccs, which differed at 14/24 loci from OMVT-1, exhibited the highest degree of shared variants to an OMV formulation ( Figure 8b). The maximum number of shared variants was 14 (n=40), and all isolates shared ≥1 variant. The lower overall number of variant matches was because most isolates shared the loci that are independent of cc, and additionally only a small number of cc-specific variants. Another way to broaden potential vaccine coverage would be to create a vaccine containing multiple OMVTs, for example a formulation representing the five most prevalent hyperinvasive ccs (OMVTs: 160, 368, 1152, 27, and 21). With this formulation all isolates in the reference set shared ≥2 variants with the proposed vaccine, with 3310/3506 (94.4%) isolates having ≥8 variant matches, across all ccs, except cc11 ( Figure 8c). All cc11 isolates had ≤8 shared variants with the multiple OMVT vaccine; however, they are highly uniform and the development of a single-strain cc11 OMV vaccine, using for example OMVT-1149, would provide good coverage ( Figure 8d), similar to that demonstrated by previously developed OMV vaccines in Cuba, Norway, and New Zealand.

## Discussion

The diversity of the meningococcus and the complexity of its infection biology ^39^, in combination with fears of the possible harmful effects of vaccines containing the serogroup B capsular antigen ^40^, have hindered the development of a universal meningococcal childhood vaccine that comprises one or a few components ^12^. The meningococcus is an ‘accidental’ pathogen, as disease plays no part in onward transmission and, indeed, is inimical to it ^39^. While disease rates are relatively low, asymptomatic carriage rates can be very high, especially in young adults in high-income settings ^41^ and interruption of transmission is an important element of the efficacy of conjugate polysaccharide vaccines ^42^. OMV vaccines have been used a number of times, with evidence for efficacy against disease caused by the outbreak strain in Cuba ^43^, Chile ^44^, Norway ^45^, New Zealand ^46^ and France ^47^. Evidence for the efficacy of OMV vaccines against carriage is less compelling. Although Bexsero ^®^ was designed as a four-component vaccine ^20^, the inclusion of the MeNZB™ OMV ^48^ complicates assessment of its impact. At the time of writing, the vaccine was undergoing post-implementation investigation in the UK ^18, 19, 49^ and evaluation of its impact on carriage in Australia ^50^. The OMVT scheme was devised and evaluated to assist in the assessment of the diversity of OMV vaccine components.

The majority of the OMVT scheme components were OMPs, constituting approximately 60% of the total protein content ^51^ known to be present in the meningococcal outer membrane, and therefore potentially exposed to diversifying selection imposed by immune responses, although the most diverse components Pil and Opa were too variable to be included in the scheme ^52^. Consistent with this view, the cytoplasmic proteins present in the OMV formulation, three of which were ribosomal subunit proteins, were the least diverse; however, among the OMPs, there was a wide range of diversity. The least diverse proteins, a putative periplasmic protein (NEISp1063) and FbpA, (NEISp0578), are likely not to have surface-exposed regions and might also be influenced by functional constraints. In the most diverse proteins, TbpA (NEISSp1690), PorA (NEISSp1364) and PorB (NEISSp2020), peptide sequence variability was localised in particular ‘variable regions’ (VRs), shown in a number of studies to represent surface-exposed parts of the sequence subject to immune attack by bactericidal antibodies ^36– 38^. As our knowledge and understanding of the OMV proteome grows, in terms of understanding protein function, location and immunological relevance to disease and cross-protection, the OMVT scheme could be amended to remove or add relevant antigens.

As reported previously for a number of meningococcal protein antigens, the variable components of the OMVT were non-randomly distributed among isolates, with the most diverse antigens exhibiting a non-overlapping structure ^53, 54^. The persistence of particular non-random, non-overlapping combinations of PorA and FetA antigens, a few of which attain high frequency in the population ^55^, has been used as evidence to support the existence of stable stain types in the meningococcal population driven by immune and/or metabolic selection ^53, 54^. This structuring was especially exhibited by the more diverse proteins in the OMVT scheme, with the exception of TbpA (NEISSp1690) and LbpA (NEISSp1468). Thus, although there were a very large number of variants of individual proteins and combinations of these variants, they grouped into a relatively small number of clusters, similar to those observed with FetA and PorA ^55^ and the BAST scheme ^23^. In addition, as with PorA:FetA types ^9^ and BASTs ^24^, OMVT clusters were strongly associated with ccs and capsular group. Ccs correspond to lineages within the meningococcal population, several of which have an increased propensity to cause invasive disease. These are referred to as the hyperinvasive lineages ^39^ and they dominate collections of isolates from IMD, although they are rarer in isolates obtained from asymptomatic carriage ^9^. A number of mechanisms have been proposed to explain the existence of persistence of these population structures in the highly recombinogenic meningococcus ^56^, but whatever the mechanism that generates them, these associations can be exploited in vaccine formulation, potentially simplifying it ^10^.

Indexing of the variation in the OMVTs in the context of representative isolate collections, such as the MRF-MGL ^33^, along with the analysis tools embedded within the PubMLST.org website, enabled the *in silico* assessment of the likely performance of different OMV formulations. The OMVT from a given isolate could be rapidly determined and the likely content of an OMV vaccine made from the isolate deduced. The number of exact protein matches present in a given meningococcal population to this vaccine could then be readily established, indicating the likely degree of coverage that vaccine might attain. This analysis only considered exact matches to vaccine OMP variants as cross-protection is not well characterised for antigens beyond PorA and FetA. Analysis of the UK reference dataset showed that the MeNZB™ OMV exhibited a similar number of exact matches to a putative cc11 OMV, with higher levels of exact matches attainable by alternative vaccine formulations or the inclusion of multiple OMVs in a single vaccine formulation. In the absence of evidence for very broad cross-protection, these comparisons suggest that multi-component or ‘designer’ OMVs that contained artificial repertoires of OMPs could have substantial advantages in terms of vaccine coverage, though this requires an understanding of regional meningococcal epidemiology with ongoing surveillance and the additional lipoproteins present in native OMV preparations.

Using a similar approach as the previously described MLST ^6^ and BAST ^23^ schemes, the OMVT scheme represents a portable and scalable means of cataloguing variation in the OMV components of meningococcal vaccines ^12^. This provides a stable basis for the comparison of the extensive variation of these molecules. The OMVT scheme has the potential to be enhanced with: (i) bioinformatic approaches that predict B- and T-cell epitopes ^57^; and (ii) phenotypic expression and immunological data, to provide a more sophisticated prediction of likely cross-protection provided by these vaccines ^58^; however, while a peptide sequence catalogue can be comprehensive, this is unlikely to be the case for phenotypes and some deduction from sequence data is likely to remain essential for the foreseeable future. Correlation of the OMVT with immune responses in infants and those that affect carriage in older individuals is of particular importance in assessing the cost-effectiveness of novel meningococcal vaccines ^18^. The embedding of the OMVT scheme within the PubMLST.org/nesseria database ^59^ enables the scheme to be implemented genus-wide, permitting inter-species as well as intra-species diversity to be explored. This is of particular importance given the renewed interest in the development of novel vaccines against *Neisseria gonorrhoeae*, the gonococcus, and the possible impact of Bexsero ^®^ on gonococcal infection ^60^. Intra-species diversity is also important when considering the possible impact of such vaccines on the oropharyngeal microbiota. Finally, this approach exemplifies how WGS data can be used to support the development and evaluation of complex multicomponent vaccines against highly variable pathogens.

## Data availability

Peptide sequences generated using Nano-LC-MS/MS have been deposited to the ProteomeXchange Consortium via the PRIDE partner repository, accession number PXD011622. DOI: https://doi.org/10.6019/PXD011622.

## Supplementary material

**Supplementary Table 1. List of proteins identified from the NZ98/254 OMV component of Bexsero ^®^ identified using multi-dimensional LC-MS/MS peptide sequencing and database searching ( *N. meningitidis* MC58, Swiss-Pro FASTA release 2014_02).** Following identification, protein FASTA sequences were submitted to a web server for cellular location prediction using two tools, subCELular LOcalisation prediction ( http://cello.life.nctu.edu.tw/cello2go/) and PSORTb ^28^. Peak area of the top three most abundant peptides were used to represent the quantity of the protein identified. OM=Outer-membrane, EC=Extracellular, PP=Periplasmic, CP=Cytoplasmic, IM=Inner-membrane. Protein number marked with * were selected as part of the OMV peptide Typing (OMVT) scheme in this study. The OMV components of both NZ98/254 (MeNZB™) and Bexsero ^®^ are detergent-extracted with a subsequent loss of fHbp when compared to a native OMV composition. Analysis of the MC58 and NZ98/254 meningococcal reference genomes on PubMLST.org revealed that Opa genes are absent in these strains.
