# Supplementary material for: Typing complex meningococcal vaccines to understand diversity and population structure of key vaccine antigens
Source: Wellcome Open Res. 2019 Mar 19;3:151. Originally published 2018 Nov 29. [Version 2] doi: 10.12688/wellcomeopenres.14859.2 (PMC6338130; doi:10.12688/wellcomeopenres.14859.2)
Supplement: Supplementary file 1 [file wellcomeopenres-3-16545-s0000.tgz › e4640b17-6485-4926-a6ff-95905e8e6e6c_Supplementary_Table_1.docx]

**Supplementary Table 1.** **List of proteins identified from the NZ98/254 OMV component of Bexsero®** identified using multi-dimensional LC-MS/MS peptide sequencing and database searching (*N. meningitidis* MC58, Swiss-Pro FASTA release 2014_02). Following identification, protein FASTA sequences were submitted to a web server for cellular location prediction using two tools, subCELular LOcalisation prediction (<http://cello.life.nctu.edu.tw/cello2go/>) and PSORTb (26). Peak area of the top three most abundant peptides were used to represent the quantity of the protein identified. OM=Outer-membrane, EC=Extracellular, PP=Periplasmic, CP=Cytoplasmic, IM=Inner-membrane. Protein numbers marked with an asterisk were selected as part of the OMV peptide typing scheme in this study.

| **Protein No** | **UniProt Accession No** | **Uniprot gene No** | **Protein Name, Gene Name (GN=)** | **Predicted Location** | | **Peak Area** |
| --- | --- | --- | --- | --- | --- | --- |
|  |  |  |  | **CELLO** | **PSORTb** |  |
| 1* | P30690 | NMB2039 | Major outer membrane protein P.IB GN=porB | OM | OM | 2.745E11 |
| 2* | P0A0V3 | NMB0382 | Outer membrane protein class 4 GN=rmpM | OM | OM | 1.325E11 |
| 3* | P0DH58 | NMB1429 | Major outer membrane protein P.IA GN=porA | OM | OM | 9.031E10 |
| 4* | Q7DDH4 | NMB1126/NMB1164 | Lipoprotein NMB1126/NMB1164 GN=NMB1126 | OM. EC, PP | Unknown | 3.275E10 |
| 5* | Q7DDI3 | NMB1053 | Class 5 outer membrane protein GN=opc | OM | OM | 2.584E10 |
| 6* | Q7DDM2 | NMB0663 | Outer membrane protein NspA GN=nspA | OM | OM | 2.377E10 |
| 7* | Q7DDB6 | NMB1497 | TonB-dependent receptor NMB1497 GN=NMB1497 | OM | OM | 1.880E10 |
| 8* | Q9JXL3 | NMB1988 | Iron-regulated outer membrane protein FrpB GN=frpB | OM | OM | 1.546E10 |
| 9 | Q7DDT2 | NMB0152 | 50S ribosomal protein L14 GN=rplN | CP, IM | CP | 1.523E10 |
| 10* | P0A0Y4 | NMB0634 | Major ferric iron-binding protein GN=fbpA | PP | PP | 1.480E10 |
| 11 | P64027 | NMB0124/NMB0139 | Elongation factor Tu GN=tufA | CP | CP | 1.469E10 |
| 12* | Q9JZN9 | NMB0964 | TonB-dependent receptor NMB0964 GN=NMB0964 | OM, EC, | Unknown | 1.152E10 |
| 13 | Q9K1I2 | NMB0161 | 50S ribosomal protein L15 GN=rplO | CP, IM | CP | 9.638E9 |
| 14 | P66355 | NMB0166 | 30S ribosomal protein S11 GN=rpsK | CP | CP | 8.959E9 |
| 15 | Q9JY71 | NMB1710 | Glutamate dehydrogenase GN=gdhA | CP | Unknown | 8.171E9 |
| 16* | Q70M91 | NMB1812 | Type IV pilus biogenesis and competence protein PilQ GN=pilQ | OM | OM | 8.153E9 |
| 17 | P05431 | NMB0018 | Fimbrial protein GN=pilE | EC, OM, PP | Extracellular | 8.130E9 |
| 18 | P66540 | NMB2101 | 30S ribosomal protein S2 GN=rpsB | CP | CP | 8.114E9 |
| 19 | Q9K1I4 | NMB0154 | 50S ribosomal protein L5 GN=rplE | CP | CP | 7.524E9 |
| 20 | P66577 | NMB0159 | 30S ribosomal protein S5 GN=rpsE | CP | CP | 7.358E9 |
| 21 | P66508 | NMB0463 | 30S ribosomal protein S20 GN=rpsT | CP  IM | CP | 6.653E9 |
| 22 | Q9K1I1 | NMB0169 | 50S ribosomal protein L17 GN=rplQ | CP, IM | CP | 6.454E9 |
| 23* | Q9K0U9 | NMB0461 | Transferrin-binding protein 1 GN=tbp1 | OM | OM | 6.424E9 |
| 24 | P66333 | NMB0140 | 30S ribosomal protein S10 GN=rpsJ | CP | CP | 6.380E9 |
| 25 | P66519 | NMB1950 | 30S ribosomal protein S21 GN=rpsU | CP. IM | CP | 6.259E9 |
| 26 | P66408 | NMB0155 | 30S ribosomal protein S14 GN=rpsN | CP. IM | CP | 6.104E9 |
| 27 | Q9K0K5 | NMB0589 | 50S ribosomal protein L19 GN=rplS | CP | CP | 6.011E9 |
| 28 | P66551 | NMB0148 | 30S ribosomal protein S3 GN=rpsC | CP, IM | CP | 5.855E9 |
| 29 | P66375 | NMB0136 | 30S ribosomal protein S12 GN=rpsL | CP | CP | 5.836E9 |
| 30 | Q9K1I3 | NMB0157 | 50S ribosomal protein L6 GN=rplF | CP, IM | CP | 5.793E9 |
| 31* | Q9JS44 | NMB0375/NMB0652 | Adhesin MafA GN=mafA1 | OM, EP | Unknown | 5.713E9 |
| 32 | Q7DDT4 | NMB0149 | 50S ribosomal protein L16 GN=rplP | CP | CP | 5.666E9 |
| 33 | Q9K0N4 | NMB0554 | Chaperone protein DnaK GN=dnaK | CP | CP | 5.561E9 |
| 34* | Q9K1F0 | NMB0204 | Outer membrane protein assembly factor BamE GN=bamE | IM, PP | OM | 5.348E9 |
| 35* | Q7DDE8 | NMB1124/NMB1162 | Lipoprotein NMB1124/NMB1162 GN=NMB1124 | OM, EP | Unknown | 5.343E9 |
| 36 | P66642 | NMB2056 | 30S ribosomal protein S9 GN=rpsI | CP | CP | 5.273E9 |
| 37 | P0A0X7 | NMB1321 | 30S ribosomal protein S18 GN=rpsR | CP, IM | CP | 5.066E9 |
| 38 | Q7DDK4 | NMB0946 | Peroxiredoxin 2 family protein/glutaredoxin GN=NMB0946 | CP, IM | Unknown | 4.902E9 |
| 39 | Q9K1I8 | NMB0138 | Elongation factor G GN=fusA | CP | CP | 4.888E9 |
| 40 | Q9K093 | NMB0723 | 50S ribosomal protein L20 GN=rplT | CP | CP | 4.867E9 |
| 41 | Q9JYI2 | NMB1574 | Ketol-acid reductoisomerase (NADP(+)) GN=ilvC | CP | CP | 4.764E9 |
| 42 | Q7DDQ8 | NMB0378 | Phosphate transporter GN=NMB0378 | IM | CPMembrane | 4.700E9 |
| 43 | Q7DDR4 | NMB0325 | 50S ribosomal protein L21 GN=rplU | CP | CP | 4.527E9 |
| 44 | Q7DDT0 | NMB0158 | 50S ribosomal protein L18 GN=rplR | CP | CP | 4.514E9 |
| 45 | P61057 | NMB0143 | 50S ribosomal protein L4 GN=rplD | CP, IM, PP | CP | 4.474E9 |
| 46 | Q9K1I6 | NMB0144 | 50S ribosomal protein L23 GN=rplW | CP, IM | CP | 4.433E9 |
| 47* | Q9K1H0 | NMB0182 | Outer membrane protein assembly factor BamA GN=bamA | OM | OM | 4.294E9 |
| 48 | Q7DDT5 | NMB0147 | 50S ribosomal protein L22 GN=rplV | CP | CP | 4.235E9 |
| 49* | Q9JZ20 | NMB1333 | Uncharacterized protein NMB1333 GN=NMB1333 | OM, IM, EP | OM | 4.073E9 |
| 50 | Q7DDH5 | NMB1125/NMB1163 | Uncharacterized protein GN=NMB1125 | CP, IM, PP | Unknown | 4.072E9 |
| 51 | P42385 | NMB1972 | 60 kDa chaperonin GN=groL | CP | CP | 3.721E9 |
| 52 | P66386 | NMB0165 | 30S ribosomal protein S13 GN=rpsM | CP, PP | CP | 3.613E9 |
| 53* | Q9K1M2 | NMB0088 | Outer membrane protein NMB0088 GN=NMB0088 | OM, EP, PP | OM | 3.498E9 |
| 54 | Q7DDS7 | NMB0171 | Septum site-determining protein MinD GN=minD | CP | CP | 3.489E9 |
| 55* | Q9K1K7 | NMB0109 | Uncharacterized protein GN=NMB0109 | IM, CP, EP | Unknown | 3.331E9 |
| 56* | Q9JY68 | NMB1714 | Multidrug efflux pump channel protein GN=mtrE | OM | OM | 3.247E9 |
| 57 | Q7DDS9 | NMB0160 | 50S ribosomal protein L30 GN=rpmD | CP, IM | CP | 3.169E9 |
| 58 | Q9JYD2 | NMB1643 | Translation initiation factor IF-2 GN=infB | CP | CP | 3.148E9 |
| 59 | Q7DDL5 | NMB0763 | Cysteine synthase GN=cysK | CP | CP | 3.134E9 |
| 60 | Q9JZN0 | NMB0980 | NAD(P) transhydrogenase subunit alpha GN=pntA | IM | IM | 3.087E9 |
| 61 | Q7DD49 | NMB2057 | 50S ribosomal protein L13 GN=rplM | CP | CP | 3.045E9 |
| 62 | P66561 | NMB0167 | 30S ribosomal protein S4 GN=rpsD | CP | CP | 2.929E9 |
| 63* | Q06379 | NMB1540 | Lactoferrin-binding protein A GN=lbpA | OM | OM | 2.928E9 |
| 64 | P65235 | NMB0875 | Ribose-phosphate pyrophosphokinase GN=prs | CP, IM | CP | 2.789E9 |
| 65 | P66628 | NMB0156 | 30S ribosomal protein S8 GN=rpsH | CP | CP | 2.726E9 |
| 66 | Q9K1I5 | NMB0145 | 50S ribosomal protein L2 GN=rplB | CP, IM | CP | 2.689E9 |
| 67 | P64371 | NMB1583 | Imidazoleglycerol-phosphate dehydratase GN=hisB | CP | CP | 2.550E9 |
| 68 | Q9JZQ2 | NMB0944 | 5-methyltetrahydropteroyltriglutamate--homocysteine methyltransferase GN=metE | CP | CP | 2.525E9 |
| 69 | Q9K1J3 | NMB0127 | 50S ribosomal protein L11 GN=rplK [RL11_NEIMB] | CP, IM, PP | CP | 2.525E9 |
| 70 | Q9K0K8 | NMB0586 | Adhesin GN=NMB0586 | PP | IM | 2.494E9 |
| 71 | Q7DDD7 | NMB1220 | Stomatin/Mec-2 family protein GN=NMB1220 | CP, IM | CP | 2.435E9 |
| 72 | P0A0S6 | NMB0427 | Cell division protein FtsZ GN=ftsZ | CP | CP | 2.426E9 |
| 73 | P66047 | NMB0130 | 50S ribosomal protein L10 GN=rplJ | CP, IM | CP | 2.414E9 |
| 74* | Q9JYI8 | NMB1567 | Probable FKBP-type peptidyl-prolyl cis-trans isomerase FkpA | OM, PP | OM | 2.367E9 |
| 75 | Q9K0B1 | NMB0703 | Outer membrane protein assembly factor BamD GN=bamD | OM, PP | OM | 2.353E9 |
| 76 | P64389 | NMB1230 | DNA-binding protein HU-beta GN=hupB | CP, IM | CP | 2.273E9 |
| 77 | Q9K0A4 | NMB0711 | Uncharacterized protein GN=NMB0711 | OM, PP | CP | 2.224E9 |
| 78 | Q9JZ44 | NMB1301 | 30S ribosomal protein S1 GN=rpsA | CP | CP | 2.135E9 |
| 79 | Q7DD63 | NMB1946 | Lipoprotein GN=NMB1946 | IM, PP | IM | 2.120E9 |
| 80 | P66088 | NMB0128 | 50S ribosomal protein L1 GN=rplA | CP | CP | 2.103E9 |
| 81 | P66614 | NMB0137 | 30S ribosomal protein S7 GN=rpsG | CP | CP | 2.081E9 |
| 82 | Q9JY02 | NMB1808 | PilM protein GN=pilM | CP, EP, OM | Unknown | 2.013E9 |
| 83 | P0DH59 | NMB1445 | Protein RecA GN=recA | CP | CP | 1.994E9 |
| 84 | Q9K0C2 | NMB0692 | Tpc protein GN=tpc | CP | Unknown | 1.905E9 |
| 85* | Q9K0U7 | NMB0464 | Phospholipase A1 GN=NMB0464 | OM, PP | OM | 1.869E9 |
| 86 | Q9JXQ0 | NMB1936 | ATP synthase subunit alpha GN=atpA | CP, IM | CP | 1.842E9 |
| 87 | Q9JZW3 | NMB0876 | 50S ribosomal protein L25 GN=rplY | CP, IM, PP | CP | 1.835E9 |
| 88 | Q9JZ37 | NMB1313 | Trigger factor GN=tig | CP | CP | 1.783E9 |
| 89 | Q7DDK0 | NMB0954 | Citrate synthase GN=gltA | CP | CP | 1.763E9 |
| 90 | Q9JZ12 | NMB1341 | Pyruvate dehydrogenase E1 component GN=pdhA | CP | CP | 1.687E9 |
| 91 | Q7DDU1 | NMB0052 | Twitching motility protein PilT GN=pilT-1 | CP, IM | CP | 1.655E9 |
| 92 | Q9JZP6 | NMB0956 | Dihydrolipoyllysine-residue succinyltransferase component of 2-oxoglutarate dehydrogenase complex GN=sucB | IM | CP | 1.647E9 |
| 93 | Q9JYA8 | NMB1668 | Hemoglobin receptor GN=hmbR | OM | OM | 1.639E9 |
| 94 | Q9K0D4 | NMB0678 | Tryptophan synthase alpha chain GN=trpA | CP | CP | 1.625E9 |
| 95 | Q9JZR5 | NMB0928 | Uncharacterized protein NMB0928 GN=NMB0928 | CP, IM, PP | IM | 1.611E9 |
| 96 | P60733 | NMB0153 | 50S ribosomal protein L24 GN=rplX | CP | CP | 1.588E9 |
| 97 | Q9JXL6 | NMB1985 | Adhesion and penetration protein GN=hap | EP, OM | OM | 1.581E9 |
| 98* | Q9K187 | NMB0280 | LPS-assembly protein LptD GN=lptD | OM | OM | 1.540E9 |
| 99 | P66487 | NMB0146 | 30S ribosomal protein S19 GN=rpsS | CP, IM | CP | 1.523E9 |
| 100 | Q9JZH2 | NMB1057 | Gamma-glutamyltranspeptidase GN=ggt | EP, IM, OM, PP | PP | 1.522E9 |
| 101 | Q9K0J1 | NMB0606 | Uncharacterized protein GN=NMB0606 | CP, IM, PP | IM | 1.508E9 |
| 102 | Q9JZ29 | NMB1323 | 30S ribosomal protein S6 GN=rpsF | IM | CP | 1.494E9 |
| 103 | Q9K134 | NMB0359 | Glutamine synthetase GN=glnA | CP | CP | 1.485E9 |
| 104 | Q9K0V0 | NMB0460 | Transferrin-binding protein 2 GN=tbpB | EP, OM | Unknown | 1.395E9 |
| 105 | Q9K0X8 | NMB0426 | Cell division protein FtsA GN=ftsA | CP | CP | 1.379E9 |
| 106 | Q7DDA0 | NMB1576 | Acetolactate synthase III, small subunit GN=ilvH | EP, PP | CP | 1.319E9 |
| 107 | Q9JXN8 | NMB1953 | Stringent starvation protein A GN=sspA | CP, IM | CP | 1.296E9 |
| 108 | P0A0X1 | NMB0131 | 50S ribosomal protein L7/L12 GN=rplL | CP | Unknown | 1.294E9 |
| 109 | Q9JY66 | NMB1716 | Membrane fusion protein GN=mtrC | CP, IM, PP | IM | 1.230E9 |
| 110 | Q9K0I2 | NMB0618 | Phosphoenolpyruvate synthase GN=ppsA | CP | CP | 1.229E9 |
| 111 | Q9JY54 | NMB1735 | GTP pyrophosphokinase GN=relA | CP | CP | 1.218E9 |
| 112 | Q9JZP4 | NMB0959 | Succinate--CoA ligase [ADP-forming] subunit beta GN=sucC | CP, IM | CP | 1.215E9 |
| 113 | P66704 | NMB0168 | DNA-directed RNA polymerase subunit alpha GN=rpoA | CP, IM | CP | 1.215E9 |
| 114 | Q7DDM4 | NMB0609 | 30S ribosomal protein S15 GN=rpsO | CP | CP | 1.207E9 |
| 115 | Q9K165 | NMB0313 | TPR repeat-containing protein NMB0313 GN=NMB0313 | CP, IM, OM, PP | Unknown | 1.185E9 |
| 116 | Q9JXW3 | NMB1861 | Acetyl-CoA carboxylase, biotin carboxylase GN=accC | CP | CP | 1.174E9 |
| 117 | Q9K027 | NMB0798 | ATP-dependent zinc metalloprotease FtsH GN=ftsH | IM | IM | 1.160E9 |
| 118 | P60444 | NMB0142 | 50S ribosomal protein L3 GN=rplC | CP | CP | 1.155E9 |
| 119 | Q9JXB7 | NMB2134 | Uncharacterized protein GN=NMB2134 | OM, PP | OM | 1.150E9 |
| 120 | P66295 | NMB0941 | 50S ribosomal protein L36 2 GN=rpmJ2 | CP, IM | Unknown | 1.149E9 |
| 121 | Q9K0H1 | NMB0631 | Phosphate acetyltransferase Pta GN=NMB0631 | CP, IM | Unknown | 1.118E9 |
| 122 | Q9K0U8 | NMB0462 | Spermidine/putrescine ABC transporter, PP spermidine/putrescine-binding protein GN=potD-1 | CP | PP | 1.108E9 |
| 123 | Q7DDQ0 | NMB0476 | Uncharacterized protein GN=NMB0476 | CP | Unknown | 1.107E9 |
| 124 | Q9JXQ2 | NMB1934 | ATP synthase subunit beta GN=atpD | CP | CP | 1.090E9 |
| 125 | Q9JZN2 | NMB0978 | NAD(P) transhydrogenase subunit beta GN=pntB | CP | IM | 1.090E9 |
| 126 | Q9K1R3 | NMB0007 | Cell division ATP-binding protein FtsE GN=ftsE | CP, IM | IM | 1.085E9 |
| 127 | P66151 | NMB0321 | 50S ribosomal protein L28 GN=rpmB | IM | CP | 1.081E9 |
| 128 | Q9JYY0 | NMB1379 | Cysteine desulfurase IscS GN=iscS | CP | CP | 1.042E9 |
| 129 | Q9JZP3 | NMB0960 | Succinate--CoA ligase [ADP-forming] subunit alpha GN=sucD | CP | CP | 1.036E9 |
| 130 | Q59622 | NMB0132 | DNA-directed RNA polymerase subunit beta GN=rpoB | CP | CP | 1.010E9 |
| 131 | Q9JXB6 | NMB2135 | Uncharacterized protein GN=NMB2135 | CP, PP | Unknown | 1.010E9 |
| 132 | Q9JXD8 | NMB2095 | Adhesin complex protein GN=NMB2095 | CP, IM | Unknown | 1.007E9 |
| 133 | Q9JZG1 | NMB1070 | 2-isopropylmalate synthase GN=leuA | IM, CP | CP | 9.997E8 |
| 134 | Q9JZQ3 | NMB0943 | 5,10-methylenetetrahydrofolate reductase GN=metF | CP | CP | 9.923E8 |
| 135 | P66438 | NMB0592 | 30S ribosomal protein S16 GN=rpsP | CP | CP | 9.852E8 |
| 136 | Q9K1J1 | NMB0133 | DNA-directed RNA polymerase subunit beta' GN=rpoC | CP, IM | CP | 9.441E8 |
| 137 | Q9JX95 | NMB2159 | Glyceraldehyde-3-phosphate dehydrogenase GN=gapA-2 | CP, IM | CP | 9.268E8 |
| 138 | Q9K062 | NMB0758 | Polyribonucleotide nucleotidyltransferase GN=pnp | CP | CP | 9.190E8 |
| 139 | P66130 | NMB0324 | 50S ribosomal protein L27 GN=rpmA | CP | CP | 8.614E8 |
| 140 | Q9JXK3 | NMB1998 | Serine-type peptidase GN=NMB1998 | CP, PP | OM | 8.576E8 |
| 141 | Q9JXD7 | NMB2096 | Probable malate:quinone oxidoreductase GN=mqo | OM, PP | CP | 8.471E8 |
| 142 | Q7DDC7 | NMB1306 | Uncharacterized protein GN=NMB1306 | CP | CP | 8.421E8 |
| 143 | Q9JYM1 | NMB1518 | Acetate kinase 1 GN=ackA1 | OM, PP | CP | 8.409E8 |
| 144 | Q9K0J3 | NMB0604 | Alcohol dehydrogenase, zinc-containing GN=NMB0604 | CP, IM | CP | 8.334E8 |
| 145 | Q9JY09 | NMB1799 | S-adenosylmethionine synthase GN=metK | CP, IM, PP | CP | 8.111E8 |
| 146 | Q9K0H4 | NMB0628 | Imidazole glycerol phosphate synthase subunit HisF GN=hisF | CP, IM | CP | 7.918E8 |
| 147 | Q9JXE1 | NMB2091 | Hemolysin GN=NMB2091 | CP, PP | PP | 7.910E8 |
| 148 | Q9K0A7 | NMB0707 | LPS-assembly lipoprotein LptE GN=lptE | CP, IM | Unknown | 7.850E8 |
| 149 | Q9JY11 | NMB1796 | Uncharacterized protein GN=NMB1796 | CP | CP | 7.809E8 |
| 150 | P56990 | NMB1055 | Serine hydroxymethyltransferase GN=glyA | CP, EP, IM | CP | 7.786E8 |
| 151 | Q7DDJ9 | NMB0955 | 2-oxoglutarate dehydrogenase, E1 component GN=sucA | CP, EP | CP | 7.770E8 |
| 152 | Q9JZ21 | NMB1332 | Carboxy-terminal peptidase GN=prc | CP, EP, IM | IM | 7.730E8 |
| 153 | P64347 | NMB1579 | ATP phosphoribosyltransferase GN=hisG | PP | CP | 7.711E8 |
| 154 | Q9JXR1 | NMB1921 | 3-oxoacyl-(Acyl-carrier-protein) reductase GN=fabG-2 | CP, PP | Unknown | 7.651E8 |
| 155 | Q9K0Z1 | NMB0410 | Transcriptional regulator MraZ GN=mraZ | CP | CP | 7.635E8 |
| 156 | Q7DDT3 | NMB0151 | 30S ribosomal protein S17 GN=rpsQ | CP, IM | CP | 7.556E8 |
| 157 | Q9JZ11 | NMB1342 | Acetyltransferase component of pyruvate dehydrogenase complex GN=aceF | CP | CP | 7.518E8 |
| 158 | Q9JZ80 | NMB1252 | Phosphoribosylformylglycinamidine cyclo-ligase GN=purM | CP | CP | 7.501E8 |
| 159 | Q9JYI0 | NMB1577 | Acetolactate synthase GN=ilvI | OM, PP | CP | 7.372E8 |
| 160 | P0A0U2 | NMB1302 | Integration host factor subunit beta GN=ihfB | CP | CP | 7.300E8 |
| 161 | Q9JZP5 | NMB0957 | Dihydrolipoyl dehydrogenase GN=lpdA1 | CP | CP | 7.263E8 |
| 162 | Q9K1I0 | NMB0170 | Probable septum site-determining protein MinC GN=minC | CP | CP | 7.213E8 |
| 163 | P0A0R1 | NMB1207 | Bacterioferritin A GN=bfrA | CP, EP | CP | 7.174E8 |
| 164 | P65592 | NMB0126 | Transcription termination/antitermination protein NusG GN=nusG | CP | CP | 7.135E8 |
| 165 | Q9K0D3 | NMB0679 | Acetyl-coenzyme A carboxylase carboxyl transferase subunit beta GN=accD | CP | CP | 7.104E8 |
| 166 | Q9JXZ0 | NMB1829 | TonB-dependent receptor GN=NMB1829 | CP | OM | 7.018E8 |
| 167 | Q9K063 | NMB0757 | Phosphoribosylaminoimidazole-succinocarboxamide synthase GN=purC | IM | CP | 6.985E8 |
| 168 | Q9K1B7 | NMB0247 | Uncharacterized protein GN=NMB0247 | CP | Unknown | 6.976E8 |
| 169 | Q9JZ18 | NMB1335 | CreA protein GN=creA | CP, EP, PP | Unknown | 6.960E8 |
| 170 | Q7DD78 | NMB1810 | PilO protein GN=pilO | IM, PP | IM | 6.907E8 |
| 171 | Q9JZ89 | NMB1240 | ABC transporter, ATP-binding protein GN=NMB1240 | CP | CP | 6.624E8 |
| 172 | Q9JYH8 | NMB1581 | Histidinol dehydrogenase GN=hisD | CP, IM | CP | 6.520E8 |
| 173 | Q9K1P2 | NMB0039 | Uncharacterized protein GN=NMB0039 | IM | Unknown | 6.471E8 |
| 174 | Q9K151 | NMB0336 | Enoyl-[acyl-carrier-protein] reductase [NADH] FabI GN=fabI [FABI_NEIMB] | CP, EP | IM | 6.352E8 |
| 175 | Q9K112 | NMB0387 | ABC transporter, ATP-binding protein GN=NMB0387 | CP | CP | 6.250E8 |
| 176 | Q9K0L8 | NMB0574 | Aminomethyltransferase GN=gcvT | IM, CP | Unknown | 6.236E8 |
| 177 | Q9JXM3 | NMB1975 | Transporter GN=NMB1975 | CP, IM | IM | 6.232E8 |
| 178 | Q9K1F8 | NMB0196 | Ribonuclease E GN=rne | CP, IM, PP | CP | 6.091E8 |
| 179 | Q9K064 | NMB0756 | Putative dTDP-L-rhamnose synthase GN=NMB0756 | CP | CP | 5.990E8 |
| 180 | Q7DDR1 | NMB0333 | Pilus assembly protein PilG GN=pilG | CP | IM | 5.843E8 |
| 181 | Q9K0G7 | NMB0638 | UTP--glucose-1-phosphate uridylyltransferase GN=galU | CP, IM | CP | 5.836E8 |
| 182 | Q7DDP9 | NMB0477 | Uncharacterized protein GN=NMB0477 | CP | Unknown | 5.766E8 |
| 183 | Q9JYY7 | NMB1368 | ATP-dependent RNA helicase GN=NMB1368 | CP, PP | CP | 5.702E8 |
| 184 | Q9K177 | NMB0295 | Signal recognition particle protein GN=ffh | CP, IM, PP | IM | 5.687E8 |
| 185 | Q9JXV5 | NMB1869 | Fructose-bisphosphate aldolase GN=cbbA | CP | CP | 5.669E8 |
| 186 | Q9JZ50 | NMB1291 | Ribonucleoside-diphosphate reductase GN=nrdA | CP, IM | CP | 5.612E8 |
| 187 | Q9K1H1 | NMB0181 | Outer membrane protein OmpH GN=NMB0181 | CP | Unknown | 5.597E8 |
| 188 | Q9JYG7 | NMB1594 | Putrescine-binding PP protein GN=potD-3 | CP | Unknown | 5.594E8 |
| 189 | Q9JXS8 | NMB1902 | DNA polymerase III subunit beta GN=dnaN | CP | CP | 5.585E8 |
| 190 | Q9K1D8 | NMB0219 | 3-oxoacyl-[acyl-carrier-protein] synthase 2 GN=fabF-1 | CP | IM | 5.550E8 |
| 191 | Q9JRV8 | NMB1139/NMB1177 | Acetyl-coenzyme A carboxylase carboxyl transferase subunit alpha GN=accA1 | CP, IM | CP | 5.476E8 |
| 192 | Q9JYK4 | NMB1541 | Lactoferrin-binding protein B GN=lbpB | EP,IM, PP | Unknown | 5.233E8 |
| 193 | P57026 | NMB1533 | Outer membrane protein H.8 GN=NMB1533 | CP | OM | 5.222E8 |
| 194 | Q9JXK6 | NMB1995 | Nitrogen regulatory protein P-II GN=glnB | CP | IM | 5.129E8 |
| 195 | P63700 | NMB1206 | Putative bacterioferritin B GN=bfrB | CP, IM | CP | 5.069E8 |
| 196 | Q9JYI4 | NMB1572 | Aconitate hydratase B GN=acnB | CP | CP | 5.048E8 |
| 197 | Q9JY36 | NMB1762 | Hemolysin activation protein HecB GN=NMB1762 | CP, IM | OM | 5.047E8 |
| 198 | Q7DDC3 | NMB1389 | RpiR/YebK/YfhH family protein GN=NMB1389 | CP | CP | 5.041E8 |
| 199 | Q9JYY4 | NMB1371 | Acetylornithine aminotransferase GN=argD ] | CP | CP | 5.017E8 |
| 200 | Q9JYD3 | NMB1642 | Transcription termination/antitermination protein NusA GN=nusA | CP | CP | 4.896E8 |
| 201 | Q9JXY2 | NMB1839 | Formate--tetrahydrofolate ligase GN=fhs | CP | CP | 4.867E8 |
| 202 | Q7DDM6 | NMB0595 | DNA-binding response regulator GN=NMB0595 | CP, IM | CP | 4.851E8 |
| 203 | Q7DDD6 | NMB1228 | Homoserine dehydrogenase GN=metM | CP | CP | 4.840E8 |
| 204 | Q7DDK9 | NMB0861 | Uncharacterized protein GN=NMB0861 | CP | Unknown | 4.802E8 |
| 205 | Q9K0D8 | NMB0671 | Malate oxidoreductase (NAD) GN=sfcA | CP | CP | 4.794E8 |
| 206 | P64100 | NMB1729 | Biopolymer transport protein ExbB GN=exbB | CP, PP | IM | 4.785E8 |
| 207 | P65533 | NMB1307 | Nucleoside diphosphate kinase GN=ndk | IM, OM, PP | Extracellular | 4.781E8 |
| 208 | P63916 | NMB0110 | Peptide deformylase GN=def | CP | CP | 4.766E8 |
| 209 | Q7DDN3 | NMB0543 | L-lactate permease GN=NMB0543 | CP | IM | 4.759E8 |
| 210 | Q9K1M4 | NMB0085 | Sodium/glutamate symporter GN=gltS | CP, IM, PP | IM | 4.702E8 |
| 211 | Q9JZV2 | NMB0888 | Uncharacterized protein GN=NMB0888 | CP | Unknown | 4.656E8 |
| 212 | Q9K169 | NMB0307 | Phospho-2-dehydro-3-deoxyheptonate aldolase GN=aroG | CP | CP | 4.531E8 |
| 213 | Q9JYQ8 | NMB1472 | Chaperone protein ClpB GN=clpB | CP | CP | 4.528E8 |
| 214 | Q9JZB5 | NMB1201 | Inosine-5'-monophosphate dehydrogenase GN=guaB | CP | CP | 4.519E8 |
| 215 | P95379 | NMB0178 | Acyl-[acyl-carrier-protein]--UDP-N-acetylglucosamine O-acyltransferase GN=lpxA | CP | CP | 4.515E8 |
| 216 | Q9K0P0 | NMB0546 | Alcohol dehydrogenase, propanol-preferring GN=adhP | CP | CP | 4.495E8 |
| 217 | Q9JYR2 | NMB1468 | Uncharacterized protein GN=NMB1468 | CP | PP | 4.438E8 |
| 218 | Q9K152 | NMB0335 | 2,3,4,5-tetrahydropyridine-2,6-dicarboxylate N-succinyltransferase GN=dapD | CP | CP | 4.433E8 |
| 219 | Q9JXW8 | NMB1855 | Carbamoyl-phosphate synthase large chain GN=carB | CP | Unknown | 4.361E8 |
| 220 | Q9JYJ8 | NMB1554 | CTP synthase GN=pyrG | EP, IM, OM | CP | 4.336E8 |
| 221 | Q9JS34 | NMB1154/NMB1192 | Sulfate adenylyltransferase GN=cysD-2 | IM, PP | CP | 4.329E8 |
| 222 | P64051 | NMB2102 | Elongation factor Ts GN=tsf | CP | CP | 4.327E8 |
| 223 | Q7DDQ9 | NMB0355 | Lipopolysaccharide export system protein LptA GN=lptA | CP | Extracellular | 4.280E8 |
| 224 | Q9JYY2 | NMB1373 | Ribosome-binding factor A GN=rbfA [RBFA_NEIMB] | CP | CP | 4.215E8 |
| 225 | Q9JZW4 | NMB0874 | 4-diphosphocytidyl-2-C-methyl-D-erythritol kinase GN=ispE | CP, IM | Unknown | 4.176E8 |
| 226 | Q9JYN6 | NMB1498 | Aspartokinase GN=lysC | CP | CP | 4.147E8 |
| 227 | Q9JYS0 | NMB1457 | Transketolase GN=tktA | CP | CP | 4.131E8 |
| 228 | Q9JZ95 | NMB1231 | Lon protease GN=lon | CP | CP | 4.129E8 |
| 229 | Q9JZV1 | NMB0889 | Uncharacterized protein GN=NMB0889 | CP | Unknown | 4.089E8 |
| 230 | P67029 | NMB1932 | Glycine--tRNA ligase alpha subunit GN=glyQ | CP, IM, PP | CP | 4.039E8 |
| 231 | P65915 | NMB1874 | Orotate phosphoribosyltransferase GN=pyrE | CP, IM | CP | 3.887E8 |
| 232 | Q9JZB7 | NMB1199 | GTP-binding protein TypA GN=typA | CP | IM | 3.864E8 |
| 233 | P57004 | NMB1730 | Protein TonB GN=tonB | CP | Unknown | 3.834E8 |
| 234 | Q9K0I3 | NMB0617 | Transcription termination factor Rho GN=rho | CP | CP | 3.818E8 |
| 235 | Q4W563 | NMB1809 | PilN protein GN=pilN | CP | IM | 3.818E8 |
| 236 | P65932 | NMB2103 | Uridylate kinase GN=pyrH | CP, IM | CP | 3.772E8 |
| 237 | Q9JYY3 | NMB1372 | ATP-dependent Clp protease ATP-binding subunit ClpX GN=clpX | CP | CP | 3.766E8 |
| 238 | Q9K135 | NMB0356 | ABC transporter, ATP-binding protein GN=NMB0356 | CP | CP | 3.748E8 |
| 239 | Q9JXT2 | NMB1897 | Leucine--tRNA ligase GN=leuS | CP | CP | 3.740E8 |
| 240 | Q9K0B8 | NMB0696 | Amino acid ABC transporter, ATP-binding protein GN=NMB0696 | CP, IM, OM, PP | IM | 3.730E8 |
| 241 | Q9K022 | NMB0804 | NAD(P)H nitroreductase GN=NMB0804 | CP | Unknown | 3.718E8 |
| 242 | Q9JS61 | NMB1150/NMB1188 | Dihydroxy-acid dehydratase GN=ilvD1 | CP | CP | 3.685E8 |
| 243 | Q9JYG6 | NMB1595 | Alanine--tRNA ligase GN=alaS | CP, IM | CP | 3.675E8 |
| 244 | Q9JYT7 | NMB1437 | Uncharacterized protein NMB1437 GN=NMB1437 | CP | CP | 3.671E8 |
| 245 | Q9JZ14 | NMB1595 | Proline--tRNA ligase GN=proS | CP | CP | 3.613E8 |
| 246 | Q9K1N4 | NMB0051 | Twitching motility protein GN=NMB0051 | PP | CP | 3.490E8 |
| 247 | Q9K1E8 | NMB0207 | Glyceraldehyde-3-phosphate dehydrogenase GN=gapA-1 | IM | CP | 3.475E8 |
| 248 | Q9K0I4 | NMB0615 | Ammonium transporter GN=NMB0615 | CP | IM | 3.467E8 |
| 249 | Q9K035 | NMB787 | Amino acid ABC transporter, PP amino acid-binding protein GN=NMB0787 | CP | PP | 3.427E8 |
| 250 | Q9JY67 | NMB1715 | Multiple transferable resistance system protein MtrD GN=mtrD | CP, IM, PP | IM | 3.383E8 |
| 251 | Q9K0H2 | NMB0630 | Imidazole glycerol phosphate synthase subunit HisH GN=hisH | CP | CP | 3.380E8 |
| 252 | Q9JZX9 | NMB0854 | Histidine--tRNA ligase GN=hisS | CP | CP | 3.367E8 |
| 253 | Q9JZ31 | NMB1320 | 50S ribosomal protein L9 GN=rplI | CP | CP | 3.290E8 |
| 254 | Q9K1Q3 | NMB0017 | UDP-3-O-acyl-N-acetylglucosamine deacetylase GN=lpxC | CP | CP | 3.230E8 |
| 255 | Q9JZW1 | NMB0878 | L-threonine dehydratase GN=ilvA | CP, IM | CP | 3.223E8 |
| 256 | Q9K147 | NMB0341 | TspA protein GN=NMB0341 | CP | OM | 3.215E8 |
| 257 | Q9JXS9 | NMB1900 | Polyphosphate kinase GN=ppk | CP, IM, PP | IM | 3.162E8 |
| 258 | P65113 | NMB0163 | Translation initiation factor IF-1 GN=infA | CP, IM | CP | 3.148E8 |
| 259 | Q9JZF7 | NMB1074 | Acetylglutamate kinase GN=argB | CP, IM | CP | 3.138E8 |
| 260 | Q9JYK7 | NMB1538 | RNA polymerase sigma factor RpoD GN=rpoD | CP, IM | CP | 3.104E8 |
| 261 | Q7DD99 | NMB1580 | Uncharacterized protein GN=NMB1580 | CP, IM | CP | 3.075E8 |
| 262 | Q9K0Z9 | NMB0401 | Bifunctional protein PutA GN=putA | IM, PP | CP | 3.074E8 |
| 263 | Q9K1H7 | NMB0174 | Valine--tRNA ligase GN=valS | CP, IM | CP | 3.057E8 |
| 264 | Q9JYN9 | NMB1493 | Carbon starvation protein A GN=cstA | CP | IM | 3.045E8 |
| 265 | Q9K103 | NMB0396 | Nicotinate-nucleotide pyrophosphorylase GN=nadC | CP, IM, PP | CP | 3.022E8 |
| 266 | Q9K1M1 | NMB0089 | Pyruvate kinase GN=pykA | CP | CP | 3.021E8 |
| 267 | Q9K0X7 | NMB0428 | Uncharacterized protein GN=NMB0428 | CP | IM | 3.019E8 |
| 268 | Q9K1P6 | NMB0035 | Efem/EfeO family lipoprotein NMB0035 GN=NMB0035 | CP | PP | 3.019E8 |
| 269 | Q9JZ53 | NMB1285 | Enolase GN=eno | IM | CP | 3.002E8 |
| 270 | Q9JXC1 | NMB2129 | Argininosuccinate synthase GN=argG | CP, IM | CP | 2.987E8 |
| 271 | Q9K0U3 | NMB0468 | Biosynthetic arginine decarboxylase GN=speA | IM, OM | Unknown | 2.965E8 |
| 272 | Q9JY94 | NMB1685 | D-lactate dehydrogenase GN=ldhA | CP, EP | CP | 2.944E8 |
| 273 | Q9JXS7 | NMB1903 | Chromosomal replication initiator protein DnaA GN=dnaA | CP | CP | 2.928E8 |
| 274 | Q9JXB2 | NMB2139 | Uncharacterized protein GN=NMB2139 | IM | Unknown | 2.924E8 |
| 275 | Q9JXX4 | NMB1849 | Carbamoyl-phosphate synthase small chain GN=carA | CP, IM | CP | 2.902E8 |
| 276 | Q9JXW9 | NMB1854 | Uncharacterized protein GN=NMB1854 | CP, IM | CP | 2.869E8 |
| 277 | Q9JY01 | NMB1814 | 3-dehydroquinate synthase GN=aroB | CP, IM | CP | 2.785E8 |
| 278 | Q9K157 | NMB0323 | UbiH family protein GN=NMB0323 | CP | Unknown | 2.770E8 |
| 279 | Q9JXY7 | NMB1833 | Isoleucine--tRNA ligase GN=ileS | CP | CP | 2.763E8 |
| 280 | Q7DDA2 | NMB1563 | Transcriptional regulator, GntR family GN=NMB1563 | CP | CP | 2.752E8 |
| 281 | P66849 | NMB1460 | Single-stranded DNA-binding protein GN=ssb | CP | CP | 2.707E8 |
| 282 | Q9JXM8 | NMB1968 | Aldehyde dehydrogenase A GN=aldA | CP | CP | 2.704E8 |
| 283 | Q9JZV5 | NMB0885 | Replicative DNA helicase GN=dnaB | CP | CP | 2.701E8 |
| 284 | Q9JZI8 | NMB1032 | Type II restriction enzyme NlaIV GN=nlaIVR | CP | CP | 2.697E8 |
| 285 | Q9JYB6 | NMB1659 | Guanosine-3`,5`-bis(Diphosphate) 3`-pyrophosphohydrolase GN=spoT | IM | CP | 2.689E8 |
| 286 | Q9JXK4 | NMB1997 | Hydroxyacylglutathione hydrolase GN=gloB | CP | CP | 2.687E8 |
| 287 | Q9JYH0 | NMB1590 | Alkyl hydroperoxide reductase AhpD GN=NMB1590 | CP, IM | Unknown | 2.686E8 |
| 288 | Q9K0U5 | NMB0466 | Aspartate--tRNA(Asp/Asn) ligase GN=aspS | CP | CP | 2.682E8 |
| 289 | Q9K048 | NMB0774 | Uracil phosphoribosyltransferase GN=upp | OM, PP | CP | 2.678E8 |
| 290 | Q9K1Q1 | NMB0029 | Glycerate dehydrogenase GN=hprA | CP, IM | CP | 2.634E8 |
| 291 | Q9JZ73 | NMB1263 | CobW-related protein GN=NMB1263 ] | CP | CP | 2.630E8 |
| 292 | Q9JXI0 | NMB2040 | Phosphomethylpyrimidine synthase GN=thiC | CP, IM | CP | 2.628E8 |
| 293 | Q9K0L9 | NMB0573 | Transcriptional regulator, AsnC family GN=NMB0573 | CP | CP | 2.608E8 |
| 294 | Q9JRU7 | NMB1153/NMB1191 | Sulfate adenylyltransferase subunit 1 GN=cysN-2 [Q9JRU7_NEIMB] | CP | CP | 2.593E8 |
| 295 | Q9JZI7 | NMB1033 | Cytosine-specific methyltransferase GN=NMB1033 | CP | CP | 2.586E8 |
| 296 | Q7DDL8 | NMB0709 | Uncharacterized protein GN=NMB0709 | CP | IM | 2.582E8 |
| 297 | Q9K1G8 | NMB0184 | 1-deoxy-D-xylulose 5-phosphate reductoisomerase GN=dxr | CP | Unknown | 2.582E8 |
| 298 | Q9JZM7 | NMB0983 | Bifunctional purine biosynthesis protein PurH GN=purH | CP | CP | 2.581E8 |
| 299 | P57011 | NMB0045 | Signal recognition particle receptor FtsY GN=ftsY | CP | IM | 2.572E8 |
| 300 | Q9JZS1 | NMB0920 | Isocitrate dehydrogenase, NADP-dependent, monomeric type GN=icd | CP, IM | CP | 2.568E8 |
| 301 | P0C277 | NMB2077 | Bifunctional protein FolD GN=folD | CP | Unknown | 2.547E8 |
| 302 | Q9JZU5 | NMB0895 | UPF0246 protein NMB0895 GN=NMB0895 | CP | CP | 2.516E8 |
| 303 | Q9JYK8 | NMB1536 | Protein translocase subunit SecA GN=secA | CP | CP | 2.506E8 |
| 304 | Q9K013 | NMB0814 | ATP phosphoribosyltransferase regulatory subunit GN=hisZ | CP | CP | 2.503E8 |
| 305 | Q9JY06 | NMB1802 | tRNA N6-adenosine threonylcarbamoyltransferase GN=tsaD | CP | Extracellular | 2.500E8 |
| 306 | Q9K0T7 | NMB0475 | Uncharacterized protein GN=NMB0475 | CP | CP | 2.468E8 |
| 307 | Q9K1Q5 | NMB0015 | 6-phosphogluconate dehydrogenase, decarboxylating GN=gnd | CP, IM, PP | CP | 2.445E8 |
| 308 | Q9JYF0 | NMB1612 | Amino acid ABC transporter, PP amino acid-binding protein GN=NMB1612 | CP | PP | 2.433E8 |
| 309 | Q4W575 | NMB0632 | Fe(3+) ions import ATP-binding protein FbpC GN=fbpC | CP, IM | IM | 2.428E8 |
| 310 | P0A0Y8 | NMB1526 | SsrA-binding protein GN=smpB | CP | CP | 2.422E8 |
| 311 | Q9JYZ1 | NMB1364 | NH(3)-dependent NAD(+) synthetase GN=NMB1364 | CP, IM | CP | 2.411E8 |
| 312 | Q9JYM0 | NMB1519 | Thiol:disulfide interchange protein DsbD GN=dsbD | CP | IM | 2.374E8 |
| 313 | Q9K0N8 | NMB0548 | AcrA/AcrE family protein GN=NMB0548 | CP | IM | 2.367E8 |
| 314 | P0A0R9 | NMB1519 | Biopolymer transport protein ExbD GN=exbD | IM | IM | 2.351E8 |
| 315 | Q9K089 | NMB0728 | Phenylalanine--tRNA ligase beta subunit GN=pheT | CP, IM | CP | 2.343E8 |
| 316 | Q9JYZ4 | NMB1361 | Pseudouridine synthase GN=NMB1361 | CP | CP | 2.340E8 |
| 317 | Q9JY23 | NMB1779 | Hemagglutinin/hemolysin-related protein GN=NMB1779 | CP, PP | OM | 2.278E8 |
| 318 | Q9K0Q0 | NMB0535 | Glucose/galactose transporter GN=gluP | IM | IM | 2.277E8 |
| 319 | Q9K095 | NMB0720 | Threonine--tRNA ligase GN=thrS | CP, IM, OM | CP | 2.259E8 |
| 320 | P65773 | NMB0807 | NAD kinase GN=nadK | CP, IM, PP | CP | 2.246E8 |
| 321 | Q9JYX5 | NMB1384 | DNA gyrase subunit A GN=gyrA | CP | CP | 2.220E8 |
| 322 | Q9K0G2 | NMB0643 | MafB-related protein GN=NMB0643 | CP, IM | Unknown | 2.219E8 |
| 323 | Q7DDM9 | NMB0561 | Protein GrpE GN=grpE | CP | CP | 2.201E8 |
| 324 | Q9JZY1 | NMB0852 | GTPase Der GN=der [DER_NEIMB] | CP | IM | 2.189E8 |
| 325 | Q9JZ09 | NMB1344 | Dihydrolipoyl dehydrogenase GN=lpdA2 | CP, IM | CP | 2.174E8 |
| 326 | Q9JXF2 | NMB2074 | Uncharacterized protein GN=NMB2074 | CP | Unknown | 2.132E8 |
| 327 | Q9JXD2 | NMB2105 | MafB protein GN=mafB | CP | Unknown | 2.121E8 |
| 328 | Q9JXJ1 | NMB2017 | ComEA-related protein GN=NMB2017 | CP | IM | 2.096E8 |
| 329 | Q7DDN2 | NMB0550 | Thiol:disulfide interchange protein DsbC GN=dsbC | CP | PP | 2.071E8 |
| 330 | Q7DD45 | NMB2094 | Uncharacterized protein GN=NMB2094 | CP | CP | 2.042E8 |
| 331 | Q9JYQ6 | NMB1475 | Uncharacterized protein GN=NMB1475 | CP, IM | Unknown | 2.041E8 |
| 332 | Q9JRT0 | NMB1127 | Oxidoreductase, short chain dehydrogenase/reductase family GN=NMB1127 | CP | CP | 2.031E8 |
| 333 | Q9JYM8 | NMB1506 | Arginine--tRNA ligase GN=argS | CP, EP, IM, PP | CP | 1.999E8 |
| 334 | Q9JZL6 | NMB0998 | Oxidoreductase GN=NMB0998 | CP | CP | 1.970E8 |
| 335 | Q9JXR6 | NMB1916 | 3-oxoacyl-[acyl-carrier-protein] synthase 3 GN=fabH | CP | CP | 1.963E8 |
| 336 | Q9JXA0 | NMB2154 | Electron transfer flavoprotein, alpha subunit GN=etfA | CP | CP | 1.962E8 |
| 337 | Q9JS04 | NMB116/NMB1131 | Chaperone protein HscA homolog GN=hscA | CP | CP | 1.960E8 |
| 338 | Q9JYT6 | NMB1438 | Uncharacterized protein GN=NMB1438 | CP | CP | 1.936E8 |
| 339 | Q7DDI8 | NMB1035 | Uncharacterized protein GN=NMB1035 | CP | Unknown | 1.924E8 |
| 340 | Q9K1P0 | NMB0041 | ABC transporter, PP solute-binding protein GN=NMB0041 | CP, IM | PP | 1.897E8 |
| 341 | Q9JYH6 | NMB1584 | 3-hydroxyacid dehydrogenase GN=NMB1584 | CP, PP | CP | 1.897E8 |
| 342 | Q9JZ03 | NMB1351 | fmu and fmv protein GN=NMB1351 | CP | CP | 1.871E8 |
| 343 | Q9K144 | NMB0346 | Uncharacterized protein GN=NMB0346 | CP | Unknown | 1.860E8 |
| 344 | Q9JZ58 | NMB1280 | Very long chain acyl-CoA dehydrogenase-related protein GN=NMB1280 | CP | CP | 1.853E8 |
| 345 | Q9JY16 | NMB1789 | Protein-export protein SecB GN=secB | CP, IM | CP | 1.848E8 |
| 346 | Q9K0P5 | NMB0540 | Aminotransferase GN=aspC | CP | CP | 1.835E8 |
| 347 | Q9JZI6 | NMB1034 | 3-isopropylmalate dehydratase small subunit GN=leuD | CP, IM | CP | 1.828E8 |
| 348 | Q9JYZ7 | NMB1358 | Aspartyl/glutamyl-tRNA(Asn/Gln) amidotransferase subunit B GN=gatB [GATB_NEIMB] | CP | CP | 1.806E8 |
| 349 | Q9JYY8 | NMB1367 | Ribosomal RNA large subunit methyltransferase K GN=rlmK | CP, IM | CP | 1.802E8 |
| 350 | Q9K186 | NMB0281 | Chaperone SurA GN=NMB0281 | CP | IM | 1.737E8 |
| 351 | Q9K1E4 | NMB0212 | DNA gyrase subunit B GN=gyrB | CP | CP | 1.727E8 |
| 352 | Q9K1P9 | NMB0031 | Glutamine--fructose-6-phosphate aminotransferase [isomerizing] GN=glmS | CP, IM | CP | 1.720E8 |
| 353 | Q9JYC3 | NMB1652 | UPF0210 protein NMB1652 GN=NMB1652 | CP | Unknown | 1.717E8 |
| 354 | Q9K0G4 | NMB0641 | Inorganic pyrophosphatase GN=ppa | CP | CP | 1.697E8 |
| 355 | Q9K0J0 | NMB0607 | Protein translocase subunit SecD GN=secD | CP | IM | 1.693E8 |
| 356 | Q9JYE7 | NMB1616 | Phosphomethylpyrimidine kinase GN=thiD | CP | CP | 1.676E8 |
| 357 | Q9JZY2 | NMB0851 | Recombination-associated protein RdgC GN=rdgC | CP | CP | 1.668E8 |
| 358 | Q7DDK2 | NMB0951 | Succinate dehydrogenase iron-sulfur subunit GN=sdhB | CP, IM, OM | IM | 1.665E8 |
| 359 | Q9JYU6 | NMB1425 | Lysine--tRNA ligase GN=lysS | CP | CP | 1.664E8 |
| 360 | Q9JYH7 | NMB1582 | Histidinol-phosphate aminotransferase GN=hisC | CP | CP | 1.617E8 |
| 361 | Q9K1N8 | NMB0044 | Peptide methionine sulfoxide reductase MsrA/MsrB GN=msrAB | CP, IM | CP | 1.617E8 |
| 362 | Q9JZ40 | NMB1310 | 4-hydroxy-3-methylbut-2-en-1-yl diphosphate synthase (flavodoxin) GN=ispG | CP, IM | CP | 1.615E8 |
| 363 | Q9K116 | NMB0381 | Cys regulon transcriptional activator GN=cysB | CP | CP | 1.604E8 |
| 364 | Q51117 | NMB1926 | Lacto-N-neotetraose biosynthesis glycosyltransferase LgtE GN=lgtE | CP  IM | CP | 1.573E8 |
| 365 | Q9JXP1 | NMB1949 | Soluble lytic murein transglycosylase GN=NMB1949 | CP | PP | 1.563E8 |
| 366 | Q9JZ59 | NMB1279 | Membrane-bound lytic murein transglycosylase B GN=NMB1279 | CP | IM | 1.562E8 |
| 367 | Q9K041 | NMB0781 | Uroporphyrinogen decarboxylase GN=hemE | CP | CP | 1.556E8 |
| 368 | Q9K0M3 | NMB0569 | Na(+)-translocating NADH-quinone reductase subunit A GN=nqrA | CP, EP, IM, PP | CP | 1.533E8 |
| 369 | Q9K0H3 | NMB0629 | 1-(5-phosphoribosyl)-5-[(5-phosphoribosylamino)methylideneamino] imidazole-4-carboxamide isomerase GN=hisA | CP | CP | 1.530E8 |
| 370 | Q9JZL7 | NMB0997 | D-lactate dehydrogenase GN=dld | CP | IM | 1.518E8 |
| 371 | Q7DD87 | NMB1737 | Secretion protein GN=NMB1737 | CP | OM | 1.516E8 |
| 372 | Q9JYQ7 | NMB1473 | Aminotransferase, class I GN=NMB1473 | CP, PP | CP | 1.510E8 |
| 373 | Q9JYZ9 | NMB1356 | Glutamyl-tRNA(Gln) amidotransferase subunit A GN=gatA [GATA_NEIMB] | CP, IM | CP | 1.507E8 |
| 374 | Q9K053 | NMB0768 | Twitching motility protein PilT GN=pilT-2 | CP | CP | 1.503E8 |
| 375 | Q9K0B7 | NMB0697 | Ribosomal RNA small subunit methyltransferase A GN=rsmA | EP, OM, PP | CP | 1.500E8 |
| 376 | Q9JZH7 | NMB1046 | Threonine synthase GN=thrC | CP | CP | 1.490E8 |
| 377 | Q9K0I9 | NMB0610 | Polyamine-transporting ATPase GN=potA-1 | CP | IM | 1.485E8 |
| 378 | P0A0V8 | NMB0071 | Capsule polysaccharide export outer membrane protein CtrA GN=ctrA | CP | OM | 1.461E8 |
| 379 | Q9JZI5 | NMB1036 | 3-isopropylmalate dehydratase large subunit GN=leuC | CP, IM | CP | 1.444E8 |
| 380 | Q9JX99 | NMB2155 | Electron transfer flavoprotein, beta subunit GN=etfB | OM, PP | PP | 1.438E8 |
| 381 | Q9JZ77 | NMB1256 | 3,4-dihydroxy-2-butanone 4-phosphate synthase GN=ribB | CP | CP | 1.431E8 |
| 382 | Q9JXK5 | NMB1996 | Phosphoribosylformylglycinamidine synthase GN=purL | CP, IM, PP | CP | 1.411E8 |
| 383 | Q7DD46 | NMB2085 | Uncharacterized protein GN=NMB2085 | CP | CP | 1.409E8 |
| 384 | Q9K1B0 | NMB0257 | NADH-quinone oxidoreductase subunit L GN=nuoL | CP | IM | 1.405E8 |
| 385 | Q9K029 | NMB0795 | Peptidyl-tRNA hydrolase GN=pth | CP, IM | CP | 1.398E8 |
| 386 | Q9JXN3 | NMB1961 | VacJ-related protein GN=NMB1961 | CP | OM | 1.385E8 |
| 387 | Q9JZ38 | NMB1312 | ATP-dependent Clp protease proteolytic subunit GN=clpP | CP | CP | 1.381E8 |
| 388 | P0A0T5 | NMB1621 | Glutathione peroxidase homolog GN=gpxA | CP | CP | 1.374E8 |
| 389 | Q7DD70 | NMB1843 | Transcriptional regulator, MarR family GN=NMB1843 | CP | CP | 1.310E8 |
| 390 | Q9K1B8 | NMB0246 | NADH dehydrogenase I, F subunit GN=nuoF | CP, IM | CP | 1.303E8 |
| 391 | Q9JZI9 | NMB1031 | 3-isopropylmalate dehydrogenase GN=leuB | CP, IM, PP | CP | 1.287E8 |
| 392 | Q9JYR3 | NMB1467 | Exopolyphosphatase GN=ppx | CP | CP | 1.273E8 |
| 393 | Q7DDR0 | NMB0345 | Peptidylprolyl isomerase GN=NMB0345 | CP | Unknown | 1.267E8 |
| 394 | Q9JXR2 | NMB1920 | GMP synthase [glutamine-hydrolyzing] GN=guaA | CP, EP, OM, PP | CP | 1.265E8 |
| 395 | Q9JZP8 | NMB0950 | Succinate dehydrogenase flavoprotein subunit GN=sdhA | CP | IM | 1.260E8 |
| 396 | Q9JY95 | NMB1684 | Serine--tRNA ligase GN=serS | CP, EP, OM | CP | 1.258E8 |
| 397 | Q9K012 | NMB0815 | Adenylosuccinate synthetase GN=purA | CP | CP | 1.249E8 |
| 398 | Q9JZQ0 | NMB0947 | 2-oxoglutarate dehydrogenase, E3 component, lipoamide dehydrogenase GN=NMB0947 | CP | CP | 1.246E8 |
| 399 | Q9K055 | NMB0766 | Elongation factor 4 GN=lepA [LEPA_NEIMB] | EP, OM, PP | IM | 1.244E8 |
| 400 | Q7DD71 | NMB1838 | Ribosome-binding ATPase YchF GN=ychF | CP | CP | 1.241E8 |
| 401 | Q9JXQ5 | NMB1930 | Glycine--tRNA ligase beta subunit GN=glyS | IM, OM, PP | CP | 1.224E8 |
| 402 | Q7DDP7 | NMB0479 | Uncharacterized protein GN=NMB0479 | CP, IM, OM, PP | Unknown | 1.203E8 |
| 403 | P56927 | NMB1560 | Glutamine--tRNA ligase GN=glnS | CP | CP | 1.194E8 |
| 404 | Q9K002 | NMB0828 | ADP-L-glycero-D-manno-heptose-6-epimerase GN=hldD | CP | CP | 1.186E8 |
| 405 | Q9JYU4 | NMB1428 | Aminopeptidase GN=NMB1428 | CP | Unknown | 1.159E8 |
| 406 | Q7DDR2 | NMB0329 | Type IV pilus assembly protein GN=pilF | CP | CP | 1.148E8 |
| 407 | Q9JZ41 | NMB1309 | Fimbrial biogenesis and twitching motility protein GN=NMB1309 | CP | OM | 1.147E8 |
| 408 | Q7DDD1 | NMB1290 | Cytosine-specific methyltransferase GN=NMB1290 | CP, IM | CP | 1.140E8 |
| 409 | Q7DDC4 | NMB1377 | L-lactate dehydrogenase GN=lldD | CP | CP | 1.116E8 |
| 410 | Q9JXN4 | NMB1958 | Putative thioredoxin GN=NMB1958 | CP | PP | 1.113E8 |
| 411 | Q9JXG5 | NMB2061 | Phosphoenolpyruvate carboxylase GN=ppc | OM | CP | 1.105E8 |
| 412 | Q9JXX6 | NMB1846 | Iron-sulfur cluster carrier protein GN=NMB1846 | CP | CP | 1.103E8 |
| 413 | P0A0Z6 | NMB1807 | Penicillin-binding protein 1A GN=mrcA | CP | Extracellular | 1.096E8 |
| 414 | Q9JXG6 | NMB2060 | Glycerol-3-phosphate dehydrogenase [NAD(P)+] GN=gpsA | CP | CP | 1.091E8 |
| 415 | Q9K0H6 | NMB0626 | Peptide chain release factor 3 GN=prfC | CP | CP | 1.050E8 |
| 416 | Q9JRV5 | NMB1128 | Uncharacterized protein GN=NMB1128 | PP | Unknown | 1.050E8 |
| 417 | Q7DD96 | NMB1614 | Trk system potassium uptake protein TrkA GN=trkA | CP | IM | 1.039E8 |
| 418 | Q9K183 | NMB0284 | Adenylosuccinate lyase GN=purB | CP, IM | Unknown | 1.037E8 |
| 419 | Q9JYH3 | NMB1587 | Putative protease GN=NMB1587 | CP | IM | 1.031E8 |
| 420 | Q9JY30 | NMB1768 | Hemagglutinin/hemolysin-related protein GN=NMB1768 | IM, PP | OM | 1.031E8 |
| 421 | Q9K1L0 | NMB0105 | PhnO-related protein GN=NMB0105 | CP | CP | 1.027E8 |
| 422 | Q9K1E2 | NMB0214 | Oligopeptidase A GN=prlC | IM | CP | 1.008E8 |
| 423 | Q9JXE9 | NMB2078 | Uncharacterized protein GN=NMB2078 | CP | IM | 1.003E8 |
| 424 | Q7DDQ7 | NMB0380 | Transcriptional regulator, Crp/Fnr family GN=NMB0380 | CP | CP | 9.935E7 |
| 425 | Q9JYA0 | NMB1679 | tRNA/tmRNA (uracil-C(5))-methyltransferase GN=trmA | CP | CP | 9.907E7 |
| 426 | Q9JYC2 | NMB1653 | UPF0237 protein NMB1653 GN=NMB1653 | CP | Unknown | 9.805E7 |
| 427 | Q9JZI4 | NMB1037 | Glutamate--cysteine ligase GN=gshA | CP, IM | CP | 9.259E7 |
| 428 | Q9K092 | NMB0724 | Phenylalanine--tRNA ligase alpha subunit GN=pheS | CP | CP | 9.160E7 |
| 429 | Q9JX97 | NMB2157 | Putative pyrazinamidase/nicotinamidase PncA GN=NMB2157 | CP, IM | CP | 8.934E7 |
| 430 | Q9JYX2 | NMB0724 | Glucose-6-phosphate 1-dehydrogenase GN=zwf | CP | CP | 8.746E7 |
| 431 | Q9JYN5 | NMB1499 | Ribonuclease PH GN=rph | CP | CP | 8.702E7 |
| 432 | Q7DD58 | NMB1983 | Uncharacterized protein GN=NMB1983 | CP | Unknown | 8.270E7 |
| 433 | Q9K0M8 | NMB0564 | Na(+)-translocating NADH-quinone reductase subunit F GN=nqrF | CP | CP | 7.790E7 |
| 434 | Q9K0E2 | NMB0667 | Cell division protein ZipA GN=NMB0667 | CP, IM, PP | Unknown | 7.699E7 |
| 435 | Q9JYX1 | NMB1393 | Phosphogluconate dehydratase GN=edd | CP, IM | CP | 7.631E7 |
| 436 | Q9K0K6 | NMB0588 | ABC transporter, ATP-binding protein GN=NMB0588 | CP | IM | 7.563E7 |
| 437 | Q9K078 | NMB0740 | DNA repair protein RecN GN=recN | EP, IM, OM | CP | 7.389E7 |
| 438 | Q9JX94 | NMB2160 | DNA mismatch repair protein MutS GN=mutS | CP | CP | 7.341E7 |
| 439 | Q9JXQ1 | NMB1935 | ATP synthase gamma chain GN=atpG | CP | Unknown | 7.241E7 |
| 440 | P65616 | NMB0106 | Aspartate carbamoyltransferase GN=pyrB | CP, IM | CP | 7.237E7 |
| 441 | Q9K1Q0 | NMB0030 | Methionine--tRNA ligase GN=metG | CP | CP | 7.202E7 |
| 442 | Q9K0V1 | NMB0459 | Uncharacterized protein NMB0459 GN=NMB0459 | CP, PP | CP | 6.965E7 |
| 443 | Q9JZ28 | NMB1324 | Thioredoxin reductase GN=trxB | CP | Unknown | 6.925E7 |
| 444 | Q9K0Z0 | NMB0411 | Ribosomal RNA small subunit methyltransferase H GN=rsmH | CP | CP | 6.778E7 |
| 445 | Q9K025 | NMB0801 | Delta-aminolevulinic acid dehydratase GN=hemB | CP | CP | 6.205E7 |
| 446 | Q9JXS2 | NMB1909 | Maf-like protein NMB1909 GN=NMB1909 | CP | CP | 6.019E7 |
| 447 | Q9JXZ2 | NMB1827 | DNA polymerase III subunit alpha GN=dnaE | CP | CP | 5.968E7 |
| 448 | Q9K0N7 | NMB0549 | Macrolide export ATP-binding/permease protein MacB GN=macB | CP | IM | 5.924E7 |
| 449 | Q9K0G8 | NMB0637 | Argininosuccinate lyase GN=argH | CP | CP | 5.717E7 |
| 450 | Q9JZB6 | NMB1200 | Ribonuclease R GN=rnr | CP | CP | 5.475E7 |
| 451 | Q9K0C1 | NMB0693 | Folylpolyglutamate synthase/dihydrofolate synthase GN=folC | CP | CP | 5.378E7 |
| 452 | Q9JYE9 | NMB1613 | Fumarate hydratase class I GN=fumB | CP | CP | 5.164E7 |
| 453 | H2VFI5 | NMB0070 | UDP-N-acetylglucosamine 2-epimerase GN=siaA | PP | CP | 4.818E7 |
| 454 | Q9JZW5 | NMB0872 | Uncharacterized protein GN=NMB0872 | CP, IM | Unknown | 3.424E7 |
| 455 | Q9K004 | NMB0825 | Putative ADP-heptose synthase GN=NMB0825 | CP | CP | 3.413E7 |
| 456 | Q9JZL5 | NMB0999 | tRNA-dihydrouridine(16) synthase GN=dusC | CP | CP | 2.904E7 |
| 457 | Q9JZ02 | NMB1353 | Aldehyde dehydrogenase family protein GN=NMB1353 | CP, IM | CP | 2.757E7 |
| 458 | Q9K017 | NMB0810 | Transcriptional regulator, TetR family GN=NMB0810 | CP | CP | 2.643E7 |
| 459 | Q9JXP9 | NMB1937 | ATP synthase subunit delta GN=atpH | CP | CP | 2.601E7 |
| 460 | Q9K0K1 | NMB0594 | Sensor histidine kinase GN=NMB0594 | CP | IM | 1.687E7 |
| 461 | Q9JXE6 | NMB2083 | Cysteine--tRNA ligase GN=cysS | IM, OM, PP | CP | 8.152E6 |
