# Supplementary material for: Typing complex meningococcal vaccines to understand diversity and population structure of key vaccine antigens
Source: Wellcome Open Res. 2019 Mar 19;3:151. Originally published 2018 Nov 29. [Version 2] doi: 10.12688/wellcomeopenres.14859.2 (PMC6338130; doi:10.12688/wellcomeopenres.14859.2)
Supplement: Peer review history

# Peer review: Reviewer response for version 1

**DOI:** 10.21956/wellcomeopenres.16194.r34347

**Published:** 2019-01-14

**Authors:** Peter van der Ley

**Affiliations:** 1 Institute for Translational Vaccinology (Intravacc), Bilthoven, The Netherlands

The paper describes a novel typing scheme to address the population diversity of meningococcal vaccine antigens. This constitutes an important step forward, as it allows a more "holistic" approach to estimate vaccine coverage. Instead of focusing on one or a few individual antigens, using this method one can assess the overall potential contributions of multiple antigens in OMV-based vaccines.

 Minor comments

1. Considering that the total OMV proteome listed in Supplementary Table 1 has 461 proteins, the selection of just 24 candidates for the scheme seems somewhat arbitrary. It would be helpful to give more discussion on the reasons for this particular selection, and how the results could be influenced by a different selection.
2. In our experience, not all outer membrane proteins are equally susceptible to the trypsin digestion step used during sample preparation for MS analysis, and therefore the details of the method used can influence the quantification results that are obtained. Have the authors compared different methods ?
3. Some well-known vaccine antigens are missing in Supplementary Table 1, in particular the Opa proteins and fHbp. Were these left out deliberately ?
4. Figure 4 contains very interesting information on variation of individual antigens. However, it is very difficult to identify the antigens corresponding to the different lines.
5. The NZ98/254 OMV used is obtained with detergent extraction. However, other methods for OMV preparation have also been described, which avoid the use of detergent extraction. Such native OMVs have different antigen composition, especially with regards to surface-exposed lipoproteins such as the well-known vaccine antigen factor H binding protein. They would therefore require different selections of OMVT antigens. It would be helpful to discuss this in the context of hypothetical novel OMV formulations (Figure 8).

I have read this submission. I believe that I have an appropriate level of expertise to confirm that it is of an acceptable scientific standard.

# Response

**Published:** 2019-03-19

**Authors:** Charlene Rodrigues

**Affiliations:** University of Oxford, UK

We are grateful for your constructive review and we have addressed all the following points:

Minor comments 

1.    Considering that the total OMV proteome listed in Supplementary Table 1 has 461 proteins, the selection of just 24 candidates for the scheme seems somewhat arbitrary. It would be helpful to give more discussion on the reasons for this particular selection, and how the results could be influenced by a different selection.

**We have clarified this in the “OMVT scheme development and nomenclature” section:**

*A total of 25 proteins were chosen, primarily for pragmatic reasons as this was considered manageable for detailed curation within the typing scheme.The proteins represented the 20 most abundant outer membrane or periplasmic proteins identified in by Nano-LC-MS/MS with an additional five proteins chosen from the published core proteome 25 , also identified by Nano-LC-MS/MS ( Table 1). All proteins chosen for the scheme were predicted to be either outer membrane or periplasmic and therefore more likely to be OMV antigens than the cytoplasmic proteins, which were more abundant in the proteomic analysis. The cytoplasmic proteins are unlikely to be constituents of the meningococcal outer membrane and their presence in OMV samples is a probable consequence of cell lysis during detergent extraction causing cytosolic proteins to be released into the preparations.*

**We have additionally noted in the “Distribution of OMVTs in IMD and association with cc” section:**

*To determine whether a different subset of OMV proteins could alter the clustering of isolates, the same 3506 meningococcal genomes were analysed using 50 OMV proteins (OMPs, periplasmic and cytoplasmic), showing similarly strong association with clonal complex (data not shown).*

2.    In our experience, not all outer membrane proteins are equally susceptible to the trypsin digestion step used during sample preparation for MS analysis, and therefore the details of the method used can influence the quantification results that are obtained. Have the authors compared different methods ?

**We have added this to the “Proteomic OMV sample preparation” section:**

*As OMPs are not all equally susceptible to enzymatic digestion, we evaluated different sample preparation methods. The method described provided an increased number of OMPs to other studies in our laboratory and the contribution of membrane to cytoplasmic proteins was comparable to other studies.*

3.    Some well-known vaccine antigens are missing in Supplementary Table 1, in particular the Opa proteins and fHbp. Were these left out deliberately ?

**We have added this in the figure legend of Supplementary Table 1:**

*The OMV components of both NZ98/254 (MeNZB™) and Bexsero® are detergent-extracted with a subsequent loss of fHbp when compared to a native OMV composition. Analysis of the MC58 and NZ98/254 meningococcal reference genomes on PubMLST.org revealed that Opa genes are absent in these strains.*

4.    Figure 4 contains very interesting information on variation of individual antigens. However, it is very difficult to identify the antigens corresponding to the different lines.

**We have made the markers on the graph larger as well as on the legend, so it will be easier to identify which colour represents the different antigens.**

5.    The NZ98/254 OMV used is obtained with detergent extraction. However, other methods for OMV preparation have also been described, which avoid the use of detergent extraction. Such native OMVs have different antigen composition, especially with regards to surface-exposed lipoproteins such as the well-known vaccine antigen factor H binding protein. They would therefore require different selections of OMVT antigens. It would be helpful to discuss this in the context of hypothetical novel OMV formulations (Figure 8).

**We have clarified this in the figure legend of Supplementary Table 1:**

*The OMV components of both NZ98/254 (MeNZB™) and Bexsero® are detergent-extracted with a subsequent loss of fHbp when compared to a native OMV composition.*

**Additionally, the assessment of other OMV proteins has been added to the “Distribution of OMVTs in IMD and association with cc” section:**

*To determine whether a different subset of OMV proteins could alter the clustering of isolates, the same 3506 meningococcal genomes were analysed using 50 OMV proteins (OMPs, periplasmic and cytoplasmic), showing similarly strong association with clonal complex (data not shown).*

# Peer review: Reviewer response for version 1

**DOI:** 10.21956/wellcomeopenres.16194.r34348

**Published:** 2018-12-18

**Authors:** Mariagrazia Pizza, Margherita Bodini

**Affiliations:** 1 GlaxoSmithKline (GSK), Siena, Italy

This manuscript describes the development of an Outer Membrane Vesicles Peptide Typing (OMVT) scheme based on a subset of selected proteins in 3506 UK invasive isolates. The paper is of great interest, well-written, and the methodology used to define the OMVT scheme and the definition of the relative clusters are clearly described. Moreover, the paper represents a milestone in vaccine development, because it provides important insight in the assessment of vaccine impact, improvement of vaccine formulations, and evaluation of inter-species and intra-species diversity.

 Minor comments:

 - page 4 **OMVT scheme development and nomenclature **paragraph: The criteria used for the selection of five additional proteins from the core proteome, and the basis for the selection of only five should be further clarified.

 - page 7 **The proteome of the OMV from Bexsero **paragraph:  It would be worth describing the differences between the number of proteins identified from this analysis, compared to what is already published.

 - **Figure 4**: The figure would be clearer by increasing the size of the dots to enable the reader to easily identify the distribution for each single antigen. A possibility could be also to zoom in the region of the dashed line, related to the four most common variants.

 - **Figure 5**: Only results related to distribution of variation for proteins known to contain variable regions and for three most conserved proteins are shown in Figure 5. However, it would be interesting to add a comment to what happens in-between: are there other peptides containing variation patterns compatible with variable regions?

 - **Discussion** at page 16 and **Abstract**: The sentence  “[…]has been used as evidence to support the existence of stable stain types in the meningococcal population driven by immune and metabolic selection 50,51]” should read “immune and/or metabolic selection”. The same applies to the abstract: ….. “is consistent with strain structure models invoking metabolic and/or host immune selection”.

 - **Discussion**: The comparison between potential coverage of real and hypothetical vaccines is very interesting. However, it could be more appropriate to clarify a few points in the discussion section:

- The differences between antigens in which a perfect match is needed to confer protection (e.g. PorA) and antigens for which the influence that sequence  variability could have on the cross-protective ability is unknown, and should be further emphasized.
- The ability of additional antigens to confer cross-protection and not included in the OMV analysis, should be mentioned and further discussed. It would be interesting to evaluate in future how much these antigens cluster with OMVT scheme.
- The sentence on page 16 : "In the absence of evidence for a very broad cross-protection[…]",highlights the importance of this study and raises a very interesting concept related to a multi-component or “designer” OMV vaccine. It would be interesting to briefly introduce also the possible limitations that such an approach may have.

We have read this submission. We believe that we have an appropriate level of expertise to confirm that it is of an acceptable scientific standard.

# Response

**Published:** 2019-03-19

**Authors:** Charlene Rodrigues

**Affiliations:** University of Oxford, UK

We welcome your constructive review and have addressed all the points as follows:

Minor comments:

- page 4 OMVT scheme development and nomenclature paragraph: The criteria used for the selection of five additional proteins from the core proteome, and the basis for the selection of only five should be further clarified.

**We have clarified this in the “OMVT scheme development and nomenclature” section:**

*A total of 25 proteins were chosen, primarily for pragmatic reasons as this was considered manageable for detailed curation within the typing scheme.The proteins represented the 20 most abundant outer membrane or periplasmic proteins identified in by Nano-LC-MS/MS with an additional five proteins chosen from the published core proteome 25 , also identified by Nano-LC-MS/MS ( Table 1). All proteins chosen for the scheme were predicted to be either outer membrane or periplasmic and therefore more likely to be OMV antigens than the cytoplasmic proteins, which were more abundant in the proteomic analysis. The cytoplasmic proteins are unlikely to be constituents of the meningococcal outer membrane and their presence in OMV samples is a probable consequence of cell lysis during detergent extraction causing cytosolic proteins to be released into the preparations.*

- page 7 The proteome of the OMV from Bexsero paragraph:  It would be worth describing the differences between the number of proteins identified from this analysis, compared to what is already published.

**We have added the following to the "Proteome of the OMV of Bexsero®" Results section:**

*Other proteomic analysis of OMVs have found between 100-300 proteins (1-3), with an increasing number of proteins identified in the OMVs presumably due to technical advances improving the sensitivity of mass spectrometers. All studies share the finding that membrane and periplasmic proteins dominate the proteome in terms of abundance.*

1.    Lappann M, Otto A, Becher D, Vogel U. 2013. Comparative proteome analysis of spontaneous outer membrane vesicles and purified outer membranes of Neisseria meningitidis. Journal of Bacteriology 195:4425-35.

2.    Masforrol Y, Gil J, Garcia D, Noda J, Ramos Y, Betancourt L, Guirola O, Gonzalez S, Acevedo B, Besada V, Reyes O, Gonzalez LJ. 2017. A deeper mining on the protein composition of VA-MENGOC-BC(R): An OMV-based vaccine against N. meningitidis serogroup B and C. Hum Vaccin Immunother 13:2548-2560.

3.    Tani C, Stella M, Donnarumma D, Biagini M, Parente P, Vadi A, Magagnoli C, Costantino P, Rigat F, Norais N. 2014. Quantification by LC-MS(E) of outer membrane vesicle proteins of the Bexsero® vaccine. Vaccine 32:1273-9.

- Figure 4: The figure would be clearer by increasing the size of the dots to enable the reader to easily identify the distribution for each single antigen. A possibility could be also to zoom in the region of the dashed line, related to the four most common variants.

**We have made the markers on the graph larger as well as on the legend, so it will be easier to identify which colour represents the different antigens.**

- Figure 5: Only results related to distribution of variation for proteins known to contain variable regions and for three most conserved proteins are shown in Figure 5. However, it would be interesting to add a comment to what happens in-between: are there other peptides containing variation patterns compatible with variable regions?

**We have added the following text to the figure 5 legend:**

T *here were other proteins that had potential variable regions including well-described protein NEISp1963 (FetA), NEISp 1468 (LbpA) and NEISp 1428 (Ton-B dependent receptor).*

- Discussion at page 16 and Abstract: The sentence  “[…]has been used as evidence to support the existence of stable stain types in the meningococcal population driven by immune and metabolic selection 50,51]” should read “immune and/or metabolic selection”. The same applies to the abstract: ….. “is consistent with strain structure models invoking metabolic and/or host immune selection”.

**Thank you, this has been changed in the updated manuscript.**

- Discussion: The comparison between potential coverage of real and hypothetical vaccines is very interesting. However, it could be more appropriate to clarify a few points in the discussion section: 

•    The differences between antigens in which a perfect match is needed to confer protection (e.g. PorA) and antigens for which the influence that sequence  variability could have on the cross-protective ability is unknown, and should be further emphasized.

**We have added the following to the discussion:**

*This analysis only considered exact matches to vaccine OMP variants as cross-protection is not well characterised for antigens beyond PorA and FetA.*

•    The ability of additional antigens to confer cross-protection and not included in the OMV analysis, should be mentioned and further discussed. It would be interesting to evaluate in future how much these antigens cluster with OMVT scheme.

**We have amended the following to include:** ******

*As our knowledge and understanding of the OMV proteome grows, in terms of understanding protein function, location, and immunological relevance to disease and cross-protection, the OMVT scheme could be amended to remove or add relevant antigens.*

•    The sentence on page 16 : "In the absence of evidence for a very broad cross-protection[…]",highlights the importance of this study and raises a very interesting concept related to a multi-component or “designer” OMV vaccine. It would be interesting to briefly introduce also the possible limitations that such an approach may have

**We have added the following limitations of this approach:**

*In the absence of evidence for very broad cross-protection, these comparisons suggest that multi-component or ‘designer’ OMVs that contained artificial repertoires of OMPs could have substantial advantages in terms of vaccine coverage, though this requires an understanding of regional meningococcal epidemiology with ongoing surveillance and the additional lipoproteins present in native OMV preparations.*
